# Moving transmission centers of embodied forage-livestock conflicts from non-pastoral provinces to pastoral provinces in China

**DOI:** 10.1016/j.fmre.2023.04.009

**Published:** 2023-05-13

**Authors:** Mingyue Yang, Sai Liang, Haifeng Zhou, Ke Li, Zhifeng Yang

**Affiliations:** a School of Environment, Beijing Normal University, Beijing 100875, China; b Key Laboratory for City Cluster Environmental Safety and Green Development of the Ministry of Education, School of Ecology, Environment and Resources, Guangdong University of Technology, Guangzhou 510006, China

**Keywords:** Rangeland, Forage-livestock conflict, Transmission center, Supply chains, Human-environment system, Betweenness

## Abstract

Forage-livestock conflict (FLC) causes rangeland degradation and poses a threat to climate change mitigation, food security, and sustainable development. Previous studies have investigated the hotspots of *in situ* FLCs. However, region-sectors of supply chains transferring embodied FLCs are overlooked. This study identifies transmission centers of embodied FLCs in China during 2005–2015 based on multi-regional input-output analysis. The results show that transmission centers are mostly light industries, such as the “agricultural and sideline products processing” sectors of Inner Mongolia and Shandong and the “textile industry” sector of Jiangsu. Moreover, the geographical distribution of transmission centers changed during 2005–2015. In particular, several transmission centers were moving from non-pastoral provinces to pastoral provinces. This is influenced by structural changes in supply chains. The findings of this study provide deep leverage points of interventions. They can support effective productivity improvement measures and intersectoral cooperation to control FLCs, and can therefore combat rangeland degradation.

## Highlights

• Transmission centers of embodied forage-livestock conflicts (FLCs) are revealed.
• Spatial distribution of transmission centers changed during 2005–2015.
• Structural changes in supply chains influence the dynamics of transmission centers.
• Productivity improvement of transmission centers can help mitigate the FLCs.

## Graphical abstract

**Figure:**
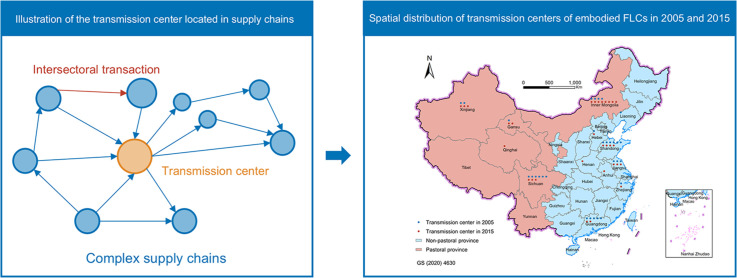

## 1 Introduction

Rangelands—extensive areas of land that are occupied by grasses, grass-like plants, forbs, or shrubs—cover approximately 54% of the Earth's surface and are grazed by domestic or wild herbivores [1]. Rangelands store vast amounts of carbon, supply and regulate water resources, and provide shelter for a great diversity of threatened wildlife [2], [3], [4]. Rangelands also support the livelihoods of billions of people and have significant cultural value [5,6]. However, nearly half of the global rangelands have been degraded [7]. Widespread rangeland degradation poses a serious threat to global food security, climate change mitigation, and sustainable development [8]. Forage-livestock conflict (FLC), which refers to the overexploitation of rangeland forage resources by herbivores, is one of the principal human drivers of rangeland degradation [9]. Therefore, the mitigation of FLCs plays an important role in combatting global rangeland degradation.

Existing studies mainly focus on the hotspots of *in situ* FLCs, including the magnitude and consequences of FLCs in rangelands and the impacts of local socioeconomic factors on FLCs. For instance, the magnitude of FLCs in rangelands has been investigated at multiple scales, including global [10,11], continental [12], national [13,14], and subnational scales [15,16]. The consequences involve ecological consequences, social consequences, and economic consequences, such as biodiversity loss [17], poverty and income inequity [18], and high additional costs of supplementary feeding [19]. The impacts of local institutional transitions and pastoralists’ decision-making on FLCs have been analysed based on field surveys [20,21]. Measures backed by these studies aim to mitigate FLCs by optimizing grazing activities in terms of setting the allowable stocking rates, regulating livestock structures, and excluding livestock grazing by using fences in extremely degraded rangelands [15,18,22].

However, according to Donella Meadows’ concept of leverage points [23], such FLC mitigation measures primarily target highly tangible but essentially weak “shallow leverage points”. According to Abson et al. [24], “shallow leverage points” are related to changes in parameters (e.g., the stocking rates) and changes in feedbacks (e.g., the time it takes for the rangeland to restore after overgrazing). These measures are easy to implement and can yield certain benefits but, on their own, are unlikely to completely address the FLC issue [24]. “Deep leverage points” are places in a complex system where relatively small interventions often lead to rather large changes in certain outcomes [25]. They are usually related to changes in system design (e.g., the structure of information flow) and changes in the intent (e.g., goals of the grazing system) [24]. In the context of global trade, the supply chains that process and transport livestock products bridge the gaps between pastoralists in rangelands and consumers worldwide [26]. The FLCs from grazing activities are also embodied in traded commodities among regions [27]. Such FLCs are known as “embodied FLCs”. There are deep leverage points hidden in supply chains. These points are usually less obvious but effective in dealing with the FLC challenge. However, the deep leverage points located in supply chains for solving the FLC challenge are yet to be fully understood.

Transmission centers located in the intermediate stages of supply chains are a major type of deep leverage point for solving the FLC challenge. Transmission centers of embodied FLCs are critical sectors that transfer substantial embodied FLCs through supply chains [28]. They usually handle the processing, manufacturing, and packaging of livestock products from producers to consumers. Transmission centers have great control over information (i.e., embodied FLCs) flowing between other sectors. Transmission centers of embodied FLCs can be identified by the node betweenness method [28]. The node betweenness method, which assesses the importance of a sector by investigating all supply chain paths that pass through it, has been widely applied by environmental researchers in recent years [29], [30], [31]. Identifying transmission centers can help propose specific control measures, such as improving utilization efficiency and formulating technical standards in these sectors [30,31]. Such transmission-bound measures can indirectly help reduce economy-wide FLCs. Therefore, it is urgent to understand the flows of embodied FLCs among sectors in complex supply chains more clearly and to seek deep leverage points for resolving the FLC challenge.

This study takes China as a case, as it has one of the largest rangelands in the world. Rangelands in China cover over 40% of the total national territory [32]. Most of the rangelands are concentrated in western China. Expansive rangelands in China are headwaters of major rivers (e.g., the Yangtze, Yellow, and Mekong rivers) and are home to millions of ethnic minorities [33]. However, approximately 70% of rangelands in China have been degraded to various extents [34]. FLC is the major human cause of rangeland degradation in China [16,35,36]. Moreover, in addition to supporting the locals, large amounts of livestock products produced in pastoral regions are traded directly or indirectly through supply chains to meet the demand of the growing urban populations [33]. Investigating the transmission centers of embodied FLCs located in complex supply chains can help mitigate FLCs in China.

To fill the above knowledge gaps and provide a better understanding of FLCs in China, this study identifies transmission centers of embodied FLCs in China based on the environmentally extended multi-regional input-output (EE-MRIO) model. We also identify critical intersectoral transactions transferring large amounts of embodied FLCs throughout supply chains. FLC is defined here as the volume of the forage gap [14]. Our findings reveal the dynamics of the transmission centers and the critical intersectoral transactions for embodied FLCs in China during 2005–2015. The identified transmission centers can offer deep leverage points for transmission-bound policy implications (e.g., improving utilization efficiency and formulating technical standards) to resolve the FLC challenge; such leverage points are usually hidden in complex supply chains and are ignored by existing studies. The results of the critical intersectoral transactions in this study can provide more explicit directions for transmission-bound FLC mitigation policy implications and intersectoral cooperation.

## 2 Materials and methods

### 2.1 EE-MRIO model

We established an EE-MRIO model to investigate the embodied FLCs associated with China's complex supply chains. The MRIO table describes the economic interdependency of different sectors [37]. By introducing environmental satellite accounts to the MRIO table, the EE-MRIO model has been widely used in analysing environmental issues, such as atmospheric mercury emissions [38,39], carbon emissions [40], deforestation [41,42], and pollutant emissions [43]. We constructed the EE-MRIO model by considering the FLCs in China as a satellite account of the Chinese subnational MRIO table. Then, we utilized the Leontief MRIO model to calculate the embodied FLCs in China driven by the final demand for products of each provincial sector. The calculation is as follows:

(1) $$c=q{\left(I-A\right)}^{-1}\hat{y}$$

(2) $$q=f{\left(\hat{x}\right)}^{-1}$$

where *c* is the FLCs in China embodied in the products of each provincial sector and ultimately purchased for the final demand; vector *q* represents the FLC intensity of each provincial sector, and the element *q_i_* can be calculated by Eq. 2; *I* is an identity matrix; *A* is the direct input coefficient matrix, of which the element *a_ij_* represents the direct input from sector *i* to produce a unitary output of sector *j*; ${\left(I-A\right)}^{-1}$ is the Leontief inverse matrix; the column vector *y* represents the final demand, of which the element *y_i_* represents the final demand for sector *i*, and $\hat{y}$ is the diagonalization of the vector *y*; the row vector *f* is the direct FLCs of each provincial sector; the elements in vector *x* represent the total output of each provincial sector; and $\hat{x}$ is the diagonalization of the vector *x*.

### 2.2 Structural path analysis

We employed structural path analysis (SPA) to track the flows of embodied FLCs along the complex supply chains. SPA is a well-established input-output based method for measuring the flows of commodities and associated environmental impacts through the supply-chain network [44]. The SPA expands the Leontief inverse matrix by using Taylor expansion, as shown in Eq. 3.

(3) $${\left(I-A\right)}^{-1}=I+A+A^{2}+A^{3}+\cdots +A^{k},\;\lim_{k\to \infty }\left(A^{k}\right)=0$$

Hence, Eq. 1 can be expressed as

(4) $$c\;=\;q\left(I+A+A^{2}+A^{3}+\cdots +A^{k}\right)y\;=\;qy+qAy+qA^{2}y+qA^{3}y+\cdots +qA^{k}y$$

Each term on the right side denotes the FLCs at a certain production layer. Different production layers contain supply chain paths with different step lengths. A supply chain path shows the environmental impacts of the starting provincial sector step by step caused by the final demand for products of the ending provincial sector. Assuming that a supply chain path starts from provincial sector *s* via *r* sectors (*k_1_, k_2_*,…,*k_r_*) and ends at provincial sector *t*, the FLCs caused by this supply chain path can be estimated by Eq. 5.

(5) $$c_{spa}=q_{s}a_{sk_{1}}a_{k_{1}k_{2}}a_{k_{2}k_{3},\cdots ,}a_{k_{r}t}y_{t}$$

where *c_spa_* represents the FLCs caused by this supply chain path; *q_s_* is the FLC intensity of the starting provincial sector *s*; $a_{sk_{1}}$, $a_{k_{1}k_{2}}$, …, $a_{k_{r}t}$ are elements in the direct input coefficient matrix *A; y_t_* represents the final demand of the ending provincial sector *t*.

### 2.3 Node betweenness

We used the node betweenness method to identify critical transmission sectors transferring embodied FLCs in China. The concept of node betweenness stems from network analysis and is defined as the amount of information passing through a node [45]. A node with a higher betweenness usually has greater control over information flowing between other nodes. It has been used in studying transportation networks [46] and social networks [47].

By considering an economy characterized by an MRIO table as a network, Liang et al. [28] introduced the node betweenness method into EE-MRIO analysis. Provincial sectors and intermediate transactions of environmental impacts between provincial sectors in an EE-MRIO model can mostly be regarded as nodes and edges in a network. The difference is that edges between nodes in most networks are undirected, while the transactions between provincial sectors in an EE-MRIO model are directed. The node betweenness of a provincial sector in an EE-MRIO analysis is measured based on the total amounts of environmental impacts of supply chain paths passing through it, as shown in Eq. 6.

(6) $$b_{i}=qTJ_{i}Ty\;$$

(7) $$T=A{\left(I-A\right)}^{-1}=A+A^{2}+A^{3}+\cdots A^{k},\;\lim_{k\to \infty }\left(A^{k}\right)=0$$

where *b_i_* represents the betweenness-based FLCs of provincial sector *i*; matrix T represents the indirect requirements for unity output of each provincial sector, calculated by Eq. 7; *J_i_* is a selection matrix, of which the (*i, i*)^th^ element is one and others are zero.

### 2.4 Edge centrality

We further used the edge centrality method to identify critical intersectoral transactions transmitting embodied FLCs in supply chains in China. The edge centrality for a particular transaction proposed by Hanaka et al. [48] indicates how much embodied environmental impacts of commodities flow through the transaction and to what extent provincial sectors are connected through the transaction. Assuming that a transaction starts from provincial sector *s* to provincial sector *t*, the edge centrality for this specific intersectoral transaction (*s, t*) is the sum of the environmental impacts associated with all supply chain paths that include this transaction, which can be calculated by Eq. 8.

(8) $$b_{st}={\left[q{\left(I-A\right)}^{-1}\right]}_{s}a_{st}x_{t}$$

where *b_st_* represents the edge centrality for a transaction from provincial sector *s* to provincial sector *t* and *x_t_* represents the total output of provincial sector *t*. More details on the stepwise derivation of the edge centrality method can be found in a previous study [48].

### 2.5 Data sources

The Chinese subnational MRIO tables were obtained from China's Industrial Ecology Virtual Laboratory (IELab) annually from 2005 to 2015 [49,50]. These original MRIO tables cover 31 provincial administrative regions (provinces, autonomous regions, and municipalities; for simplicity, they are collectively called provinces) and 42 economic sectors for each province. They are non-competitive MRIO tables. Hong Kong, Macao, and Taiwan are not covered due to data unavailability. We chose China's IELab dataset because it is the only long-term MRIO dataset that includes Tibet, the province with the greatest scope of rangelands in China. To reveal more details about rangeland-related sectors, we disaggregated the 42 sectors in the original IELab subnational table into 58 sectors based on the proxy variable vectors [51]. Details of provinces and sectors are shown in Fig. S1, Table S1, and Table S2.

We constructed the satellite account of the EE-MRIO model by using the Chinese forage-livestock conflict inventory from 2005 to 2015. The Chinese forage-livestock conflict inventory is compiled by subtracting the forage availability from the forage demand of domestic and wild herbivores in rangelands. We utilized the rangeland forage yield model to calculate the forage availability [52]. The rangeland yield model can evaluate the forage availability at the grid level, based on multiple-source remote sensing datasets such as land use maps, vegetation distribution map, and net primary production data. This model has been the main tool in assessing China's rangeland resources [14,16]. Different types of herbivores were converted into the standard sheep unit, which equals a 50 kg adult sheep that consumes 1.8 kg forage each day, to estimate the total forage demand of herbivores in rangelands based on the literature [53,54]. Detailed calculations and datasets are shown in the Supplementary Methods and Table S3. It is worth noting that only the “rangeland-based livestock raising” sectors of seven pastoral provinces (including Inner Mongolia, Sichuan, Yunnan, Tibet, Qinghai, Gansu, and Xinjiang) have FLC values in the satellite account, with FLC values of other provincial sectors being zero.

## 3 Results

### 3.1 Critical provincial sectors transmitting embodied FLCs in China

**Fig. 1:**
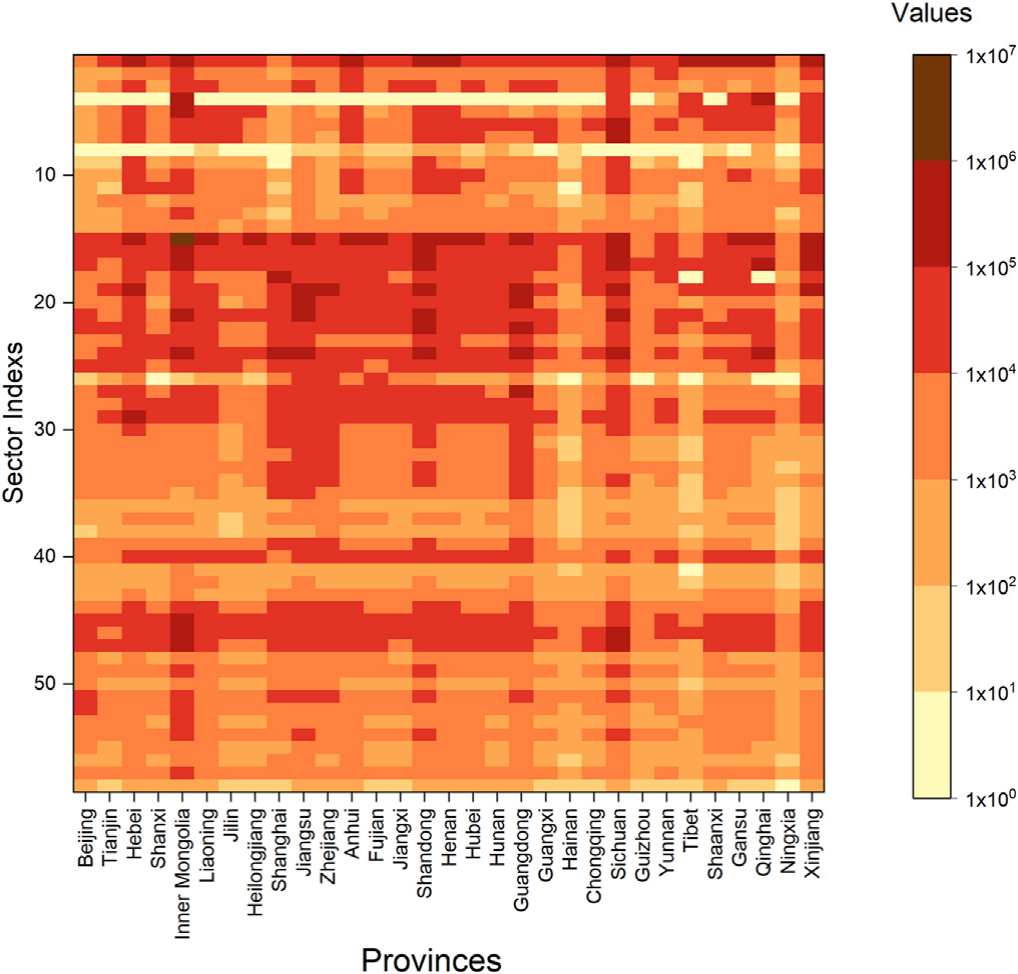
**Betweenness-based forage-livestock conflicts of provincial sectors in China in 2015.** The sector indexes and corresponding names are shown in Table S2.

Fig. 1 shows the values of betweenness-based FLCs for provincial sectors in China in 2015. The sectors with relatively high betweenness-based FLCs are mostly agriculture and light industries (e.g., the “agriculture and sideline products processing”, “food manufacturing”, and “textile industry” sectors). These provincial sectors are placed at the center of the supply chains, transmit large amounts of FLCs, and act as critical transmission sectors. The possible reason for the high betweenness-based FLCs of these sectors is that they use large quantities of upstream FLC-intensive products as intermediate inputs. For example, the “agricultural and sideline products processing” sector uses large amounts of livestock products supplied by the upstream “rangeland-based livestock raising” sector for intermediate production and then provides the processed products to downstream sectors (e.g., the “catering” and “lodging” sectors). This would result in considerable amounts of FLCs being transmitted by the “agricultural and sideline products processing” sector.

Among the 1,798 provincial sectors, the “agricultural and sideline products processing” sector of Inner Mongolia has the largest betweenness-based FLCs, transmitting 1,777,000 tons (1,777 kt) of embodied FLCs in China. It was the most critical transmission center of embodied FLCs in China in 2015. The “agricultural and sideline products processing” sector of Shandong ranks second and transmits 964 kt of embodied FLCs in China. Other critical transmission centers include the “agriculture” sector of Inner Mongolia, the “food manufacturing” sector of Inner Mongolia, and the “textile industry” sector of Jiangsu, which transmit 804, 749, and 727 kt of embodied FLCs in China, respectively. Improving the utilization efficiency of inputs from upstream sectors in these transmission centers would mitigate the FLCs induced by upstream sectors and meet the demand of downstream sectors. Fig. 1 also shows that Inner Mongolia is the most critical transmission hub influencing embodied FLCs in China. A significant number (8 out of the top 30) of transmission centers were situated in Inner Mongolia in 2015. This is consistent with Inner Mongolia's “livestock products processing and export base” role in China.

The betweenness-based FLCs of approximately 30% of provincial sectors have increased during 2005–2015 (Table S5), such as the “agriculture” sectors of Inner Mongolia and Gansu and the “beverage manufacturing” sector of Inner Mongolia. The betweenness-based FLCs of these three sectors in 2015 increased by 188 kt, 158 kt, and 126 kt than in 2005, respectively. Notably, embodied FLCs transmitted by the “agriculture, forestry, animal husbandry and fishery service” sector of Gansu have increased by more than five times during this period, from 8 kt in 2005 to 49 kt in 2015. Policymakers should pay attention to these sectors where transmission functions for embodied FLCs have increased significantly. Meanwhile, the betweenness-based FLCs of approximately 70% of provincial sectors have decreased during 2005–2015 (Table S5). These sectors are mainly distributed in non-pastoral provinces and pastoral provinces with decreasing *in situ* FLCs (i.e., Tibet and Sichuan, Table S6). In particular, embodied FLCs transmitted by the “textile industry” sector of Zhejiang and the “agriculture and sideline products processing” sectors of Shandong and Sichuan have decreased significantly during this period. The betweenness-based FLCs of these three sectors decreased by 250 kt, 246 kt, and 201 kt during 2005–2015, respectively.

**Fig. 2:**
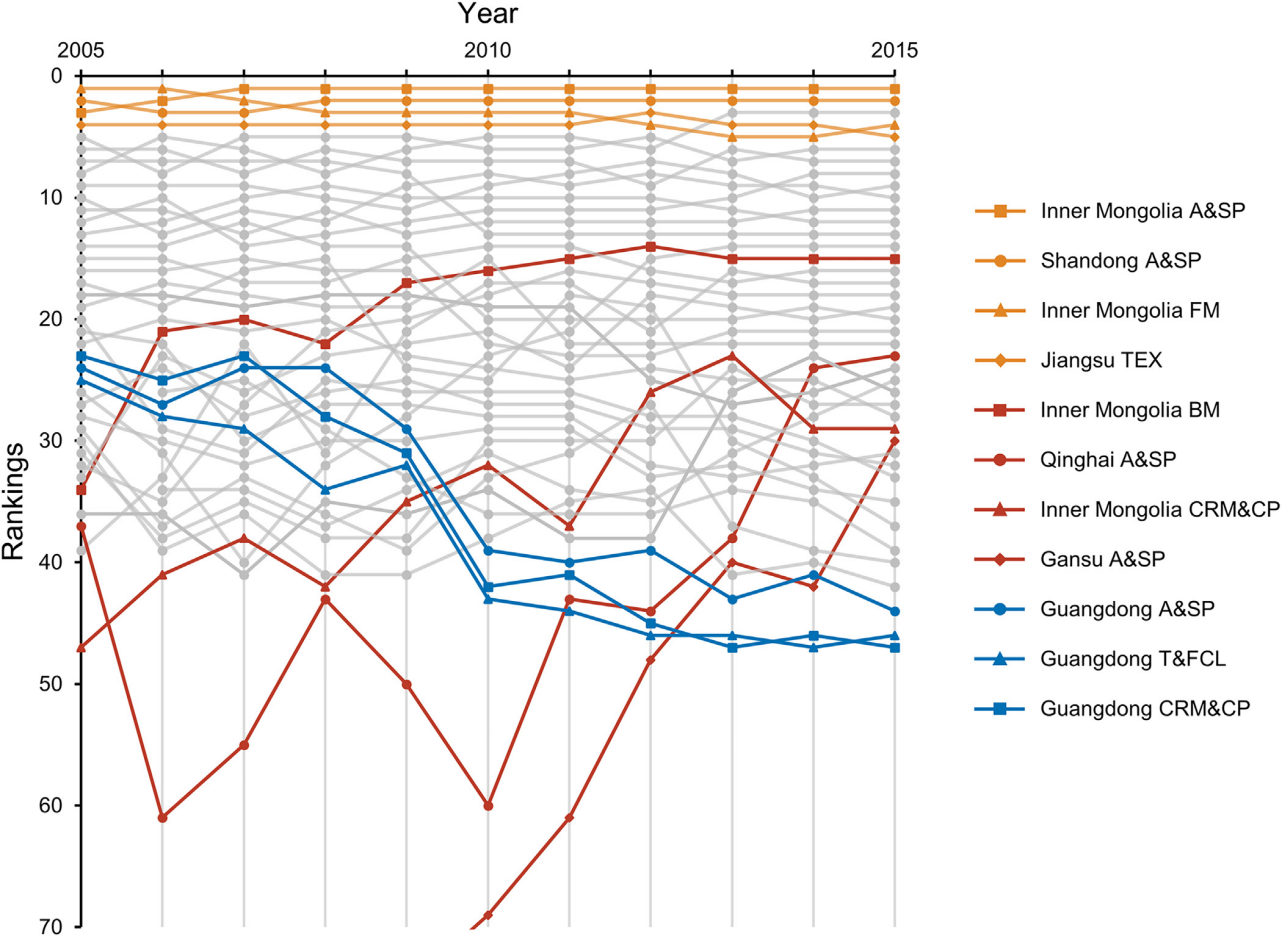
**Temporal variations in the rankings of transmission centers of embodied forage-livestock conflicts in China during 2005–2015.** The red lines show the typical rising provincial sectors, the yellow lines show the dominant transmission centers, the blue lines show the typical declining provincial sectors, and the gray lines show provincial sectors that did not change significantly. The abbreviations are shown in Table S2. The underlying data for this figure are shown in Table S4.

The rankings of some transmission centers fluctuated during 2005–2015 (Fig. 2). The rankings of several provincial sectors in terms of betweenness-based FLCs have increased during this period, suggesting that their transmission roles have become more crucial. For instance, the ranking of the “agriculture and sideline products processing” sector of Gansu in terms of betweenness-based FLCs increased from the 106th in 2006 to 30th in 2015. The amounts of embodied FLCs transmitted by this sector increased from 92 kt in 2006 to 189 kt in 2015. Another typical example is the “beverage manufacturing” sector of Inner Mongolia. The ranking of this provincial sector in terms of betweenness-based FLCs increased from 34th in 2005 to 15th in 2011 and then remained within the top 15 during 2012–2015.

In contrast, the rankings of several provincial sectors in terms of betweenness-based FLCs decreased during 2005–2015. This pattern is most obvious in the light industries sectors of Guangdong. For example, the rankings of the “chemical raw materials and chemical products”, the “agriculture and sideline products processing”, and the “textile garments, footwear, caps, leather, etc.” sectors of Guangdong in terms of betweenness-based FLCs decreased from 23rd, 24th, and 25th in 2005 to 47th, 44th, and 46th in 2015, respectively. The betweenness-based FLCs of these three sectors have decreased from 233 kt, 231 kt, and 229 kt in 2005 to 138 kt, 154 kt, and 138 kt in 2015, respectively. This indicates the diminished roles of these provincial sectors in transmitting embodied FLCs in China.

Although the rankings of several transmission centers fluctuated during 2005–2015, the dominant transmission centers remained relatively stable. These sectors are the “agricultural and sideline products processing” sectors of Inner Mongolia and Shandong, the “food manufacturing” sector of Inner Mongolia, and the “textile industry” sector of Jiangsu. These four provincial sectors ranked in the top five in terms of the betweenness-based FLCs for all study years. The average amounts of betweenness-based FLCs for these four sectors during 2005–2015 were 1529 kt, 1119 kt, 1019 kt, and 898 kt, respectively. These provincial sectors deserve major attention because they have a great influence on the flows of embodied FLCs through supply chains. Interventions targeting these provincial sectors have enormous potential to mitigate the embodied FLCs in China flowing through supply chains.

**Fig. 3:**
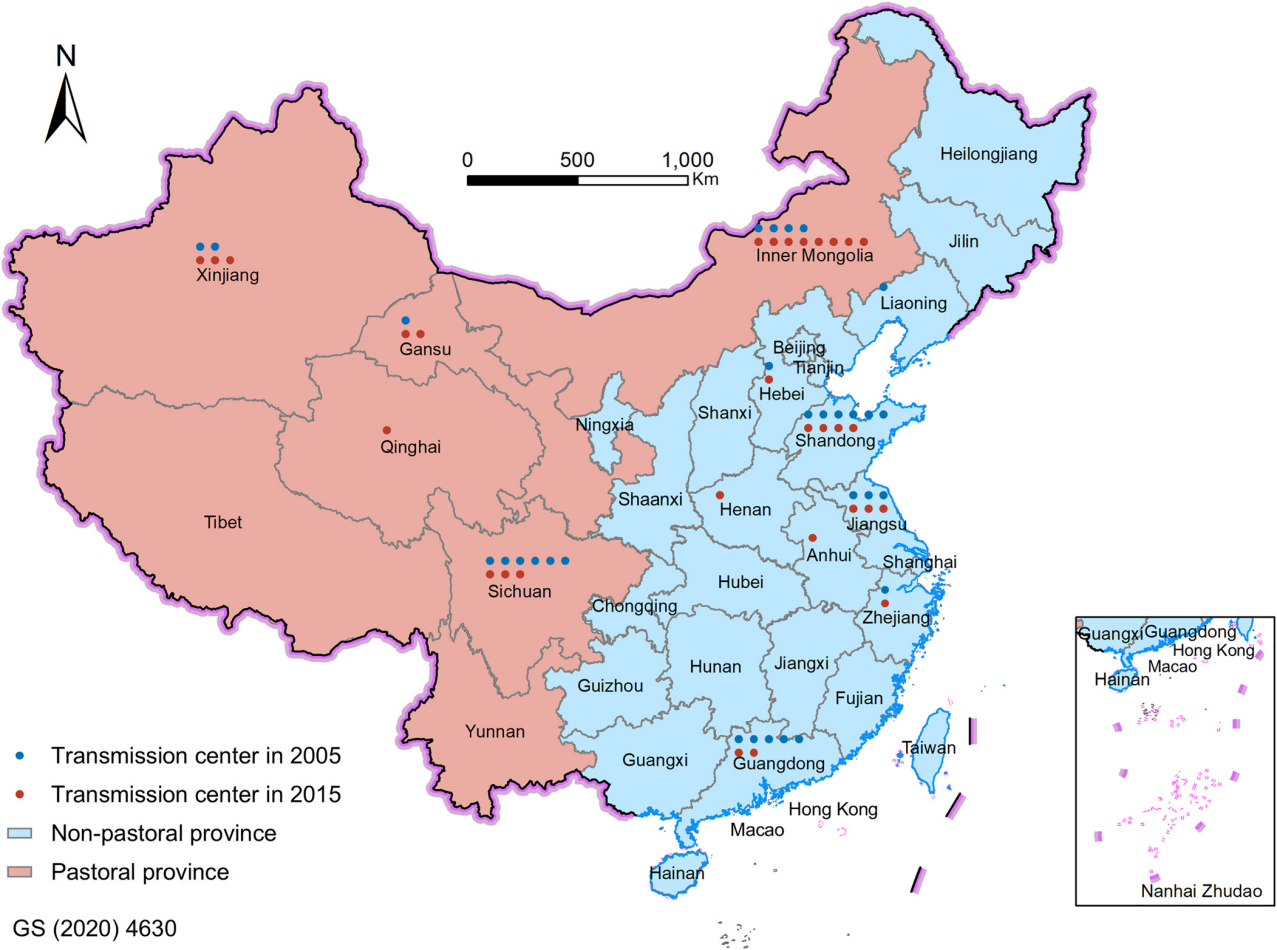
**Spatial distribution of transmission centers of embodied forage-livestock conflicts in China.** The numbers of blue and red dots represent the numbers of transmission centers in 2005 and 2015.

There were significant differences in the spatial distribution of transmission centers in different years (Fig. 3, full results in Fig. S2). In 2005, transmission centers of embodied FLCs in China were mostly located in non-pastoral provinces. For example, there were six, five, and three transmission centers in Shandong, Guangdong, and Jiangsu in 2005, respectively. However, the transmission centers moved from non-pastoral provinces to pastoral provinces during the study period. In 2015, although some transmission centers were still located in non-pastoral provinces, most transmission centers were placed in pastoral provinces. It should be noted that the number of transmission centers in Sichuan decreased from six in 2005 to three in 2015 because this province has made massive efforts to reduce its *in situ* FLCs (Table S6). The emerging transmission centers in pastoral provinces have a key role to play in mitigating economy-wide FLCs.

Moreover, we aggregated the sectoral results to obtain the betweenness-based FLCs of provinces during 2005–2015 (Table S7). We observed that the trend of betweenness-based FLCs over the decade was not the same for pastoral provinces and non-pastoral provinces (Fig. S4). The betweenness-based FLCs of pastoral provinces have increased during this period (except for Sichuan and Tibet), indicating the more and more important roles of pastoral provinces in transmitting FLCs. One of the most significant examples is Gansu. Gansu posted a 58% increase in the betweenness-based FLCs during this period, from 773 kt in 2005 to 1,224 kt in 2015. The transmission functions of Qinghai and Inner Mongolia in embodied FLCs also became more critical. The absolute values of betweenness-based FLCs of these two provinces have increased by 18% (193 kt) and 15% (789 kt) during 2005–2015, respectively. In contrast, we found that non-pastoral provinces, except for Fujian, Henan, and Ningxia, experienced a decrease in the betweenness-based FLCs during 2005–2015. Examples are Guangdong, Zhejiang, and Beijing. The betweenness-based FLCs of these three provinces have decreased by approximately 35% during this period, from 3,074 kt, 2,015 kt, and 586 kt in 2005 to 1,987 kt, 1,358 kt, and 279 kt in 2015, respectively.

### 3.2 Critical intersectoral transactions for embodied FLCs in China

The transmission centers of embodied FLCs in China recognized above can provide deep leverage points of interventions. However, for these transmission centers, implementing supply chain management strategies that focus on materials from all upstream provincial sectors can be extremely difficult. Hence, we use the edge centrality method to identify critical intersectoral transactions through which substantial embodied FLCs flow. The identified intersectoral transactions can provide transmission centers with explicit directions for the improvement of utilization efficiency.

**Fig. 4:**
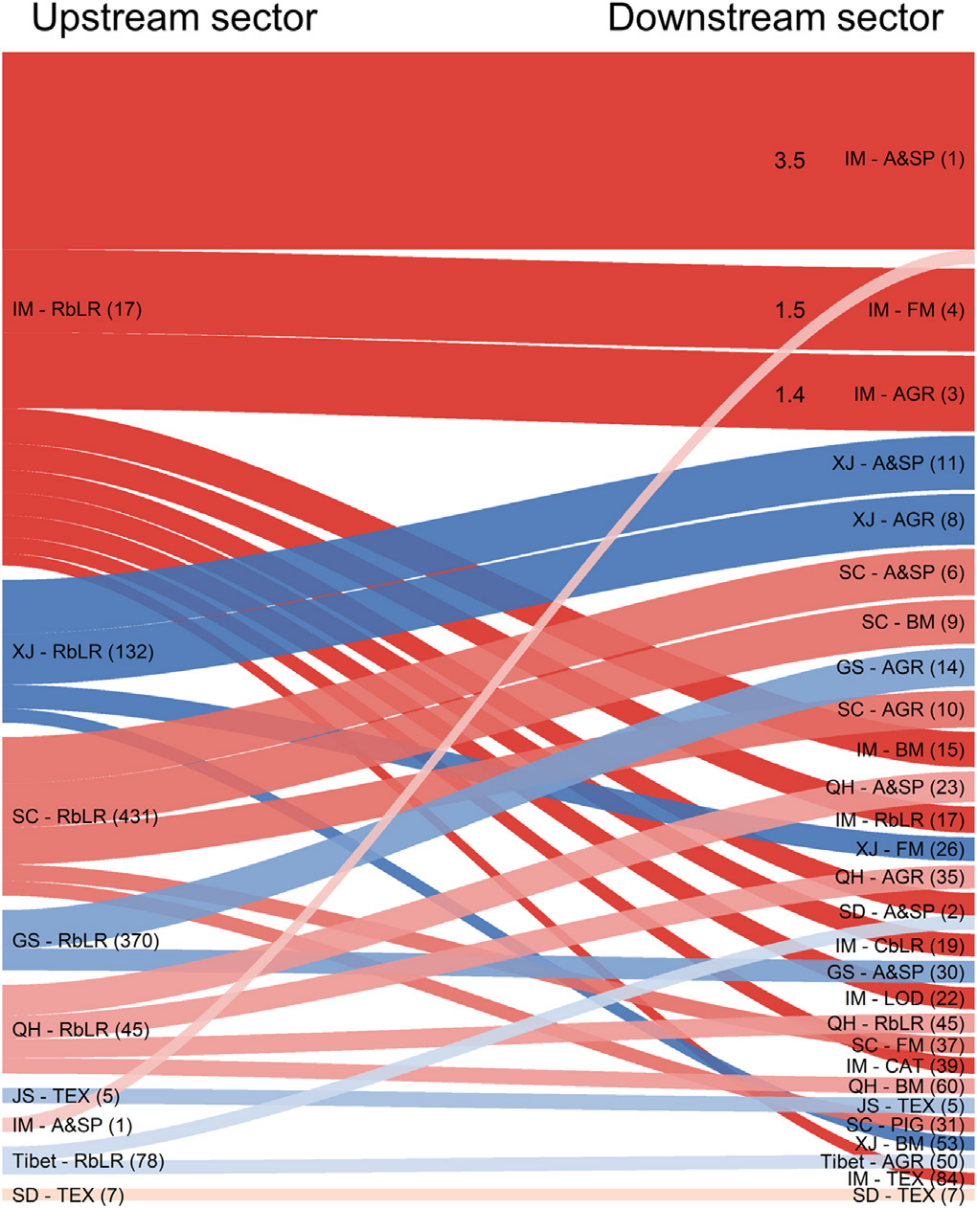
**Top 30 intersectoral transactions for embodied forage-livestock conflicts in China in 2015.** The numbers in brackets are the rankings in terms of the betweenness-based forage-livestock conflicts. The numbers marked on the lines indicate the values of edge centrality (unit: million tons, Mt). The abbreviations are shown in Table S1 and Table S2.

Fig. 4 illustrates the top 30 intersectoral transactions leading to embodied FLCs in China in 2015. These intersectoral transactions play an important role in transmitting embodied FLCs through complex supply chains. Each critical intersectoral transaction contains at least one provincial sector with relatively high betweenness-based FLCs. One of the typical examples is the transaction from the “rangeland-based livestock raising” sector of Inner Mongolia (ranking 17th in terms of betweenness-based FLCs) to its “agricultural and sideline products processing” sector (ranking 1st in terms of betweenness-based FLCs). Moreover, the crucial intersectoral transactions are mainly domestic intersectoral transactions, except for transactions from the “rangeland-based livestock raising” sectors of Inner Mongolia and Tibet to the “agricultural and sideline products processing” sector of Shandong. This demonstrates that intraregional cooperation between different sectors is of great importance to the mitigation of economy-wide FLCs. Promoting cooperation, whether technical or economic, between sectors of high edge centrality has the potential to effectively mitigate the embodied FLCs in China. The critical intersectoral transactions for embodied FLCs in China in 2015 are listed in Table S8.

**Fig. 5:**
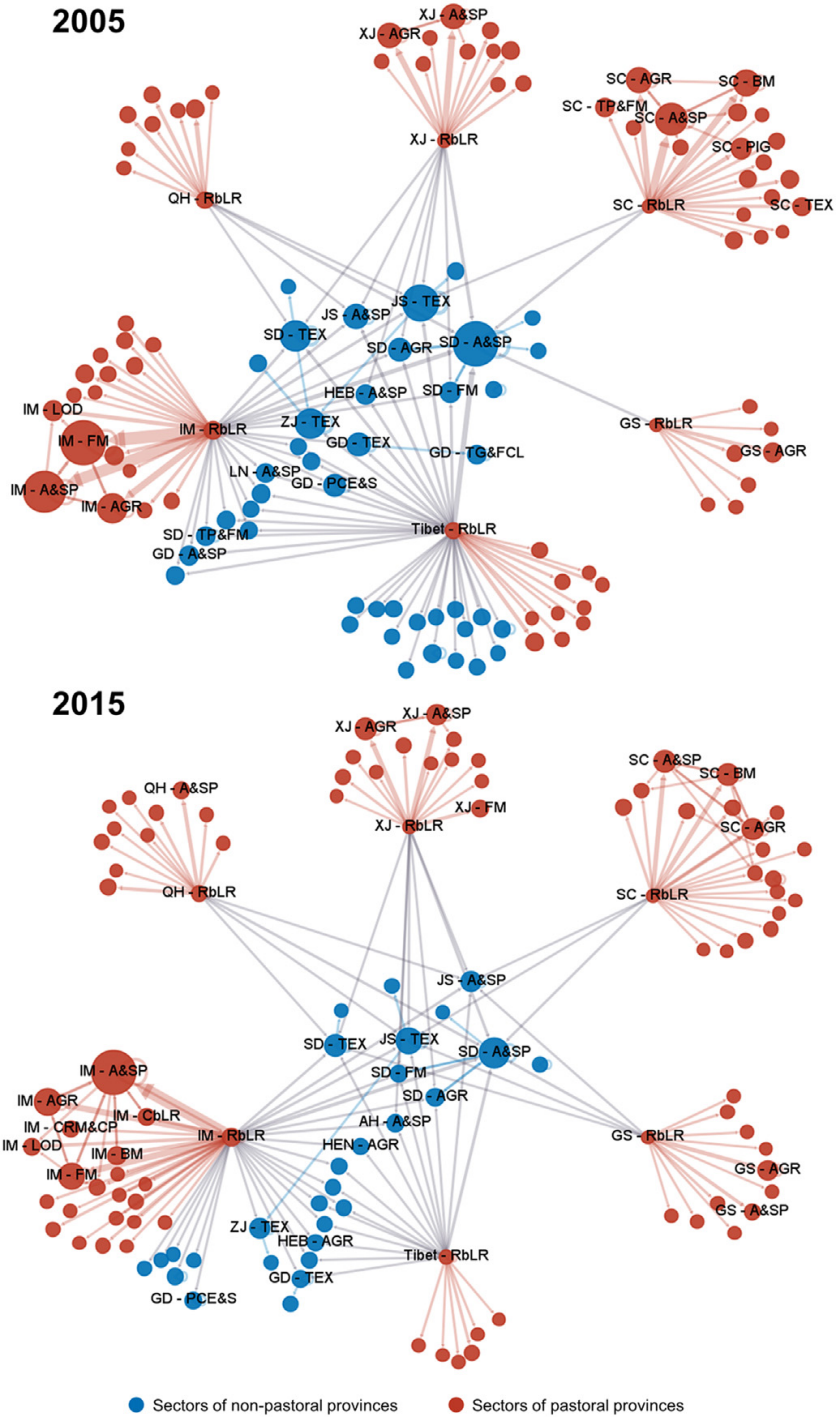
**High-priority supply chain networks with the top 200 intersectoral transactions in terms of edge centrality for embodied forage-livestock conflicts in China in 2005 and 2015.** The size of the nodes indicates the betweenness-based forage-livestock conflicts of the provincial sectors, and the width of the arrows indicates the edge centrality for embodied forage-livestock conflicts in China for the intersectoral transactions. The arrows start from the upstream provincial sectors for critical intersectoral transactions and end at their downstream provincial sectors. The abbreviations are shown in Table S1 and Table S2. The underlying data for this figure can be found in Table S8 and Table S9.

We further examine the edge centrality matrix, which contains the edge centrality value of each intersectoral transaction. Fig. 5 shows the supply chain networks composed of the top 200 intersectoral transactions in terms of edge centrality for embodied FLCs in China in 2005 and 2015. In the high-priority supply chain networks shown in this figure, a larger node indicates a higher betweenness-based FLC of a provincial sector, while a wider arrow indicates a higher edge centrality for embodied FLCs in China for an intersectoral transaction. We observe some differences in the structural characteristics between the high-priority supply chain network in 2005 and that in 2015. For example, the number of cross-border intersectoral transactions was 73 in 2005 among the top 200 intersectoral transactions for embodied FLCs in China. However, this number decreased to 58 in 2015. Meanwhile, the number of domestic intersectoral transactions in most pastoral provinces increased during this period. This trend implies a more important role of domestic intersectoral transactions in transmitting embodied FLCs in China. Moreover, among the top 200 intersectoral transactions for embodied FLCs in China, the number of critical intersectoral transactions, including the “rangeland-based livestock raising” sector of Tibet, decreased from 47 in 2005 to 23 in 2015. The structural changes in supply chains may influence the variations in transmission centers during the decade.

The structural changes in supply chains may be caused by three main factors: (1) the results of the Great Western Development Program, (2) the interregional industrial transfer from the East to the West, and (3) lower *in situ* FLCs in a few pastoral provinces. First, the “Great Western Development Program” implemented in 2000, combined with other stimulus programs, provides large amounts of investments to the least developed western pastoral provinces [55,56]. Massive investments are mainly allocated for infrastructure-related projects, including transportation and logistics, and utilities such as electricity, telecommunication, water, and sewerage [56]. Improvements in the quantity and quality of infrastructures in western pastoral provinces create conditions for the development of local industries [57]. Second, due to growing labor costs, limited land resources, rigorous environmental regulations, and requirements for industrial upgrading, some industries (especially labor-intensive manufacturing industries) in the eastern provinces of China have started transferring to other areas [58]. A substantial number of industries in eastern provinces moved to western pastoral provinces because of the relatively low costs of labor and land and relatively low tax burden in these provinces. Third, although the national *in situ* FLCs exhibited an overall increasing trend during 2005–2015, the *in situ* FLCs of several pastoral provinces decreased significantly (Table S5). This is perhaps due to the combined effect of proper weather conditions and restrictive grazing regulating policies [16]. For example, grazing activities in the “rangeland-based livestock raising” sector of Tibet induced only approximately half of the *in situ* FLCs in 2015 compared with that in 2005 (Table S5). This would undermine the role of intersectoral transactions, including this sector, in transmitting FLCs in pastoral China.

## 4 Discussion and conclusion

Previous studies on FLCs in pastoral China have focused on in situ FLCs. Measures based on these studies mainly aim to mitigate FLCs by regulating grazing activities [15,16,18,22,59]. Such measures as direct regulations on grazing activities target shallow leverage points that are easy to intervene in but are insufficient for combating China's FLC challenge. Existing studies have not addressed the deep leverage points located in supply chains influencing FLCs in pastoral China. Transmission centers that transfer large amounts of embodied FLCs through supply chains are a major part of the deep leverage points. In this study, we investigated the dynamics of transmission centers of embodied FLCs in China during 2005–2015. The results show that transmission centers are mostly light industries, such as the “agricultural and sideline products processing” sectors of Inner Mongolia and Shandong and the “textile industry” sector of Jiangsu. Geographically, some transmission centers have shifted from non-pastoral to pastoral provinces during the decade. In addition, we identified critical intersectoral transactions driving embodied FLCs in China during this period to support targeted supply chain management practices. Our findings can help policymakers formulate effective transmission-bound policy implications and support intersectoral cooperation to mitigate FLCs in China. This analytical framework is also applicable for the mitigation of FLCs in the rangelands of other regions such as Central Asia, Mongolia, and Kenya.

### 4.1 Implications

First, in the context of rising agricultural trade, interventions against FLCs in China should focus not only on shallow leverage points in the “rangeland-based livestock raising” sector but also on deep leverage points located in the intermediate parts of the supply chains. Our results show that approximately half of FLCs in China flowed among sectors through supply chains during 2005–2015 (Fig. S3). The betweenness-based results of this study can identify transmission centers of embodied FLCs in China for transmission-bound policy implications [28]. Critical transmission centers are mostly light industries, such as the “agricultural and sideline products processing” sectors of Inner Mongolia and Shandong, the “food manufacturing” sector of Inner Mongolia, and the “textile industry” sector of Jiangsu. For these transmission centers, improving the utilization efficiency is critical in resolving the FLC issue. That is, use fewer intermediate products from upstream FLC-intensive sectors to produce unitary products [60]. Potential measures for the improvement of utilization efficiency include optimizing production processes, retrofitting existing equipment, and improving staff qualifications levels [31]. Enterprises that are regarded as transmission centers would most likely welcome these measures because the improvement of utilization efficiency can help reduce their inputs and costs [30]. Moreover, since most transmission centers are related to food, reducing food loss through the design and deployment of end-to-end cold chain systems can help mitigate FLCs in China [61].

Second, emerging transmission centers in pastoral provinces deserve more attention. The results show that the betweenness-based FLCs of several sectors in pastoral provinces, such as the “agricultural and sideline products processing” sector of Qinghai, the “agriculture, forestry, animal husbandry, and fishery service” and the “agricultural and sideline products processing” sectors of Gansu, and the “catering” and the “beverage manufacturing” sectors of Inner Mongolia, increased significantly during the study period. The rising transmission role of these provincial sectors may be caused by the rapid industrialization in pastoral provinces, which results from the combined effect of the “Great Western Development Program” and other policy implications such as the “Interregional Industrial Transfer”. Rapid industrialization in pastoral provinces has led to the emergence of a large number of local enterprises, especially small, micro, and medium enterprises [62]. Local governments could formulate and apply sector-specific utilization efficiency standards for these enterprises. Enterprises that meet the highest standards can enjoy preferential treatment, while those that are below the lowest standards are required to make a thorough overhaul, or not allowed to proceed. According to the newly revised Chinese Animal Husbandry Law, livestock slaughtering and primary-processing enterprises are encouraged to move to main livestock-producing areas. Therefore, the transmission role of several light industry sectors (e.g., the “agricultural and sideline products processing” sector and the “food manufacturing” sector) in pastoral provinces is expected to become increasingly important. Improving the utilization efficiency of fast-growing light industries in pastoral provinces can help control FLCs in China effectively.

Third, cooperative mechanisms between the two ends of the critical intersectoral transactions should be established to effectively reduce embodied FLCs in China. For a transmission center, improving the utilization efficiency for all connected provincial sectors is difficult. Critical intersectoral transactions identified in this study show to what extent provincial sectors are connected through supply chains and provide specific directions for the implementation of transmission-bound measures [48]. For a critical intersectoral transaction, enterprises in the upstream sector should optimize their production activities to provide lower-FLC products to the downstream sector, while enterprises in the downstream sector should improve their utilization efficiency of products from the upstream sector. Therefore, intersectoral cooperation is needed. For example, livestock-slaughtering enterprises in Inner Mongolia (which belong to the “agricultural and sideline products processing” sector of Inner Mongolia) can develop custom equipment (e.g., mechanized slaughtering lines and mechanized carcass unloading facilities), in collaboration with enterprises in the “rangeland-based livestock raising” sector of Inner Mongolia, to increase livestock slaughtering performance, which can reduce FLCs driven by this transaction. Moreover, agricultural and sideline product processing enterprises in Inner Mongolia could provide funding to support the development of pastoralist education programs for sustainable rangeland management, which may reduce in situ FLCs induced by unsustainable grazing activities. These intersectoral cooperations should be encouraged by industry associations and local governments.

### 4.2 Limitations and uncertainties

This study has several limitations. First, the MRIO data used in this study have time lags. Compiling a more recent MRIO table can improve the applicability of policy implications. Nonetheless, we believe that some of the results of our study have reference value for policymakers today because no previous studies have revealed the deep leverage points in China's complex supply chains which could help solve the widespread FLC challenge. Second, the sectoral resolution of the MRIO data can affect the results of this study. If MRIO data with a higher sectoral resolution can be obtained, we may find new additional critical intersectoral transactions and transmission centers. Third, this study focuses on FLCs in China and pays little attention to the consequences of FLCs. Considering the consequences of FLCs as MRIO satellite accounts in future studies can help improve the understanding of the impacts of supply chain drivers on rangelands.

The majority of uncertainties stem from the calculation of in situ FLCs in China. We used the uncertainty ranges given in the Chinese forage-livestock conflict inventory to evaluate the uncertainties in this study. For example, in 2015, the ranges of embodied FLCs in China transmitted by the “agricultural and sideline products processing” sectors of Inner Mongolia and Shandong, the “food manufacturing” sector of Inner Mongolia, and the “textile industry” sector of Jiangsu are 1.4–2.2, 0.8–1.1, 0.6–0.9, and 0.6–0.8 Mt, respectively. The range of embodied FLCs in China for the transaction from the “rangeland-based livestock raising” sector of Inner Mongolia to its “agricultural and sideline products processing” sector is 2.8–4.3 Mt. Full results of the uncertainties in 2015 can be found in Table S10 and Table S11. Improving the accuracy of the Chinese forage-livestock conflict inventory would help reduce the uncertainties in this study.

## Declaration of competing interest

The authors declare that they have no conflicts of interest in this work.

## Appendix Supplementary materials

Image, application 1

Image, application 2

## References

[1] ILRI, FAO, IUCN, WWF, UNEP, ILC (2021).

[2] Bai Y., Cotrufo M.F. (2022). Grassland soil carbon sequestration: Current understanding, challenges, and solutions. Science.

[3] Liu H., Hou L., Kang N. (2022). The economic value of grassland ecosystem services: A global meta-analysis. Grassl. Res..

[4] Bengtsson J., Bullock J.M., Egoh B. (2019). Grasslands-more important for ecosystem services than you might think. Ecosphere.

[5] Godde C.M., Boone R.B., Ash A.J. (2020). Global rangeland production systems and livelihoods at threat under climate change and variability. Environ. Res. Lett..

[6] Dong S., Wolf S.A., Lassoie J.P. (2017). Bridging the gaps between science and policy for the sustainable management of rangeland resources in the developing world. Bioscience.

[7] Gang C., Zhou W., Chen Y. (2014). Quantitative assessment of the contributions of climate change and human activities on global grassland degradation. Environ. Earth Sci..

[8] Sun J., Wang Y., Piao S. (2022). Toward a sustainable grassland ecosystem worldwide. Innovation.

[9] Bardgett R.D., Bullock J.M., Lavorel S. (2021). Combatting global grassland degradation. Nat. Rev. Earth Environ..

[10] Fetzel T., Havlik P., Herrero M. (2017). Quantification of uncertainties in global grazing systems assessment. Glob. Biogeochem. Cycles.

[11] Piipponen J., Jalava M., de Leeuw J. (2022). Global trends in grassland carrying capacity and relative stocking density of livestock. Glob. Change Biol..

[12] GEOARC (2021).

[13] de Leeuw J., Rizayeva A., Namazov E. (2019). Application of the MODIS MOD 17 net primary production product in grassland carrying capacity assessment. Int. J. Appl. Earth Obs. Geoinformation.

[14] Yang T., Dong J., Huang L. (2023). A large forage gap in forage availability in traditional pastoral regions in China. Fundam. Res..

[15] Cao Y., Wu J., Zhang X. (2019). Dynamic forage-livestock balance analysis in alpine grasslands on the Northern Tibetan Plateau. J. Environ. Manage..

[16] Huang L., Ning J., Zhu P. (2021). The conservation patterns of grassland ecosystem in response to the forage-livestock balance in North China. J. Geogr. Sci..

[17] Alkemade R., Reid R.S., van den Berg M. (2013). Assessing the impacts of livestock production on biodiversity in rangeland ecosystems. Proc. Natl. Acad. Sci..

[18] Fang X., Wu J. (2022). Causes of overgrazing in Inner Mongolian grasslands: Searching for deep leverage points of intervention. Ecol. Soc..

[19] Zhang R., Yeh E.T., Tan S. (2021). Marketization induced overgrazing: The political ecology of neoliberal pastoral policies in Inner Mongolia. J. Rural Stud..

[20] Li A., Wu J., Zhang X. (2018). China’s new rural “separating three property rights” land reform results in grassland degradation: Evidence from Inner Mongolia. Land Use Policy.

[21] Yeh E.T., Samberg L.H., Gaerrang (2017). Pastoralist decision-making on the Tibetan Plateau. Hum. Ecol..

[22] Fayiah M., Dong S., Khomera S.W. (2020). Status and challenges of Qinghai–Tibet Plateau’s grasslands: An analysis of causes, mitigation measures, and way forward. Sustainability.

[23] Meadows D. (1999).

[24] Abson D.J., Fischer J., Leventon J. (2017). Leverage points for sustainability transformation. Ambio.

[25] Fischer J., Riechers M. (2019). A leverage points perspective on sustainability. People Nat..

[26] zu Ermgassen E.K.H.J., Lima M.G.B., Bellfield H. (2022). Addressing indirect sourcing in zero deforestation commodity supply chains. Sci. Adv..

[27] Qi J., Wang Y., Liang S. (2019). Primary suppliers driving atmospheric mercury emissions through global supply chains. One Earth.

[28] Liang S., Qu S., Xu M. (2016). Betweenness-based method to identify critical transmission sectors for supply chain environmental pressure mitigation. Environ. Sci. Technol..

[29] He K., Mi Z., Chen L. (2021). Critical transmission sectors in embodied atmospheric mercury emission network in China. J. Ind. Ecol..

[30] Wen W., Feng C., Zhou H. (2021). Critical provincial transmission sectors for carbon dioxide emissions in China. Renew. Sustain. Energy Rev..

[31] Yang X., Liang S., Qi J. (2021). Identifying sectoral impacts on global scarce water uses from multiple perspectives. J. Ind. Ecol..

[32] Dong S. (2022). Views on distinguishing the concepts of rangeland and grassland and proposing proper use of their terminology (in Chinese). Chin. J. Ecol..

[33] Liu M., Dries L., Huang J. (2019). The impacts of the eco-environmental policy on grassland degradation and livestock production in Inner Mongolia, China: An empirical analysis based on the simultaneous equation model. Land Use Policy.

[34] Tang F. (2021). http://www.news.cn/fortune/2021-08/20/c_1127780934.htm.

[35] The State Council (2021). http://www.gov.cn/gongbao/content/2021/content_5600082.htm.

[36] Wang Y., Lv W., Xue K. (2022). Grassland changes and adaptive management on the Qinghai–Tibetan Plateau. Nat. Rev. Earth Environ..

[37] Miller R., Blair P. (2009).

[38] Li Y., Chen L., Liang S. (2020). Spatially explicit global hotspots driving China’s mercury related health impacts. Environ. Sci. Technol..

[39] Jetashree, Zhong Q., Zhou H. (2022). Role of trade in India’s rising atmospheric mercury emissions. Environ. Sci. Technol..

[40] Xu D., Zhang Y., Chen B. (2022). Identifying the critical paths and sectors for carbon transfers driven by global consumption in 2015. Appl. Energy..

[41] Hoang N.T., Kanemoto K. (2021). Mapping the deforestation footprint of nations reveals growing threat to tropical forests. Nat. Ecol. Evol..

[42] Zhang Q., Qi J., Cheng B. (2021). Planetary boundaries for forests and their national exceedance. Environ. Sci. Technol..

[43] Yang X., Zhong Q., Liang S. (2022). Global supply chain drivers of agricultural antibiotic emissions in China. Environ. Sci. Technol..

[44] Lenzen M. (2007). Structural path analysis of ecosystem networks. Ecol. Model..

[45] Freeman L.C. (1978). Centrality in social networks conceptual clarification. Soc. Netw..

[46] Tamakloe R., Hong J., Tak J. (2021). Finding evacuation routes using traffic and network structure information. Transp. Res. Part Transp. Environ..

[47] Lee J., Lee Y., Oh S.M. (2021). Betweenness centrality of teams in social networks. Chaos Interdiscip. J. Nonlinear Sci..

[48] Hanaka T., Kagawa S., Ono H. (2017). Finding environmentally critical transmission sectors, transactions, and paths in global supply chain networks. Energy Econ..

[49] Wang Y. (2017). An industrial ecology virtual framework for policy making in China. Econ. Syst. Res..

[50] Wang Y., Geschke A., Lenzen M. (2017). Constructing a time series of nested multiregion input–output tables. Int. Reg. Sci. Rev..

[51] Liang Y., Li Y., Liang S. (2020). Quantifying direct and indirect spatial Food–Energy–Water (FEW) Nexus in China. Environ. Sci. Technol..

[52] Su D., Yang Z., Yun X. (2015).

[53] Meng N., Wang L., Qi W. (2023). A high-resolution gridded grazing dataset of grassland ecosystem on the Qinghai–Tibet Plateau in 1982–2015. Sci. Data.

[54] Dong Y., Zhao Q., Na R. (2020).

[55] Jiang M., Behrens P., Yang Y. (2022). Different material footprint trends between China and the world in 2007-2012 explained by construction- and manufacturing-associated investment. One Earth.

[56] Li P., Tian Y., Wu J. (2021). The Great Western Development policy: How it affected grain crop production, land use and rural poverty in western China, CHINA Agric. Econ. Rev..

[57] Jia J., Ma G., Qin C. (2020). Place-based policies, state-led industrialisation, and regional development: Evidence from China’s Great Western Development Programme. Eur. Econ. Rev..

[58] Yu Y., Zhang N. (2022). Does industrial transfer policy mitigate carbon emissions? Evidence from a quasi-natural experiment in China. J. Environ. Manage..

[59] Wang Z., Deng X., Song W. (2017). What is the main cause of grassland degradation? A case study of grassland ecosystem service in the middle-south Inner Mongolia. CATENA.

[60] Li H., Li K., Liang Y. (2021). Uncovering the structure of virtual multi-regional grey water network in China. Resour. Conserv. Recycl..

[61] Ren Q.-S., Fang K., Yang X.-T. (2022). Ensuring the quality of meat in cold chain logistics: A comprehensive review. Trends Food Sci. Technol..

[62] (2016).

