# Supplementary material for: Moving transmission centers of embodied forage-livestock conflicts from non-pastoral provinces to pastoral provinces in China
Source: Fundam Res. 2023 May 13;5(4):1622–30. doi: 10.1016/j.fmre.2023.04.009 (PMC12327824; doi:10.1016/j.fmre.2023.04.009)
Supplement: Supplementary file 1 [file mmc1.docx]

Supplementary Information of

**Moving transmission centers of embodied forage-livestock conflicts from non-pastoral provinces to pastoral provinces in China**

*Mingyue Yang ^1^, Sai Liang ^2*^, Haifeng Zhou ^1^, Ke Li ^2^, Zhifeng Yang ^2^*

^1^ School of Environment, Beijing Normal University, Beijing 100875, People’s Republic of China

^2^ Key Laboratory for City Cluster Environmental Safety and Green Development of the Ministry of Education, School of Ecology, Environment and Resources, Guangdong University of Technology, Guangzhou, 510006, People’s Republic of China

* Corresponding author: liangsai@gdut.edu.cn

Number of pages: 21

Number of figures: 5

Number of tables: 6

# Supplementary Figures

**
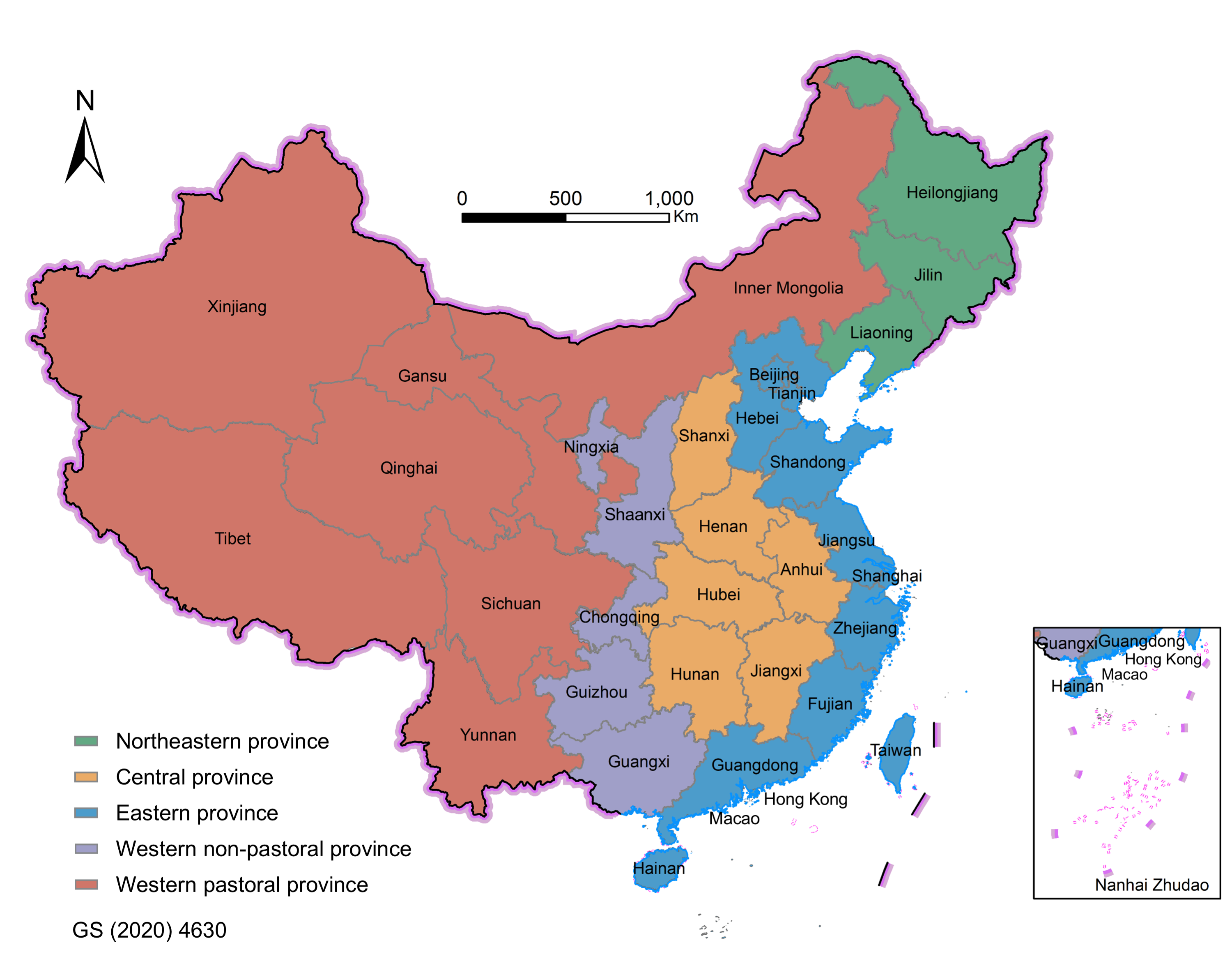
**

Fig. S1. The geographical boundary for regions and provinces in China.


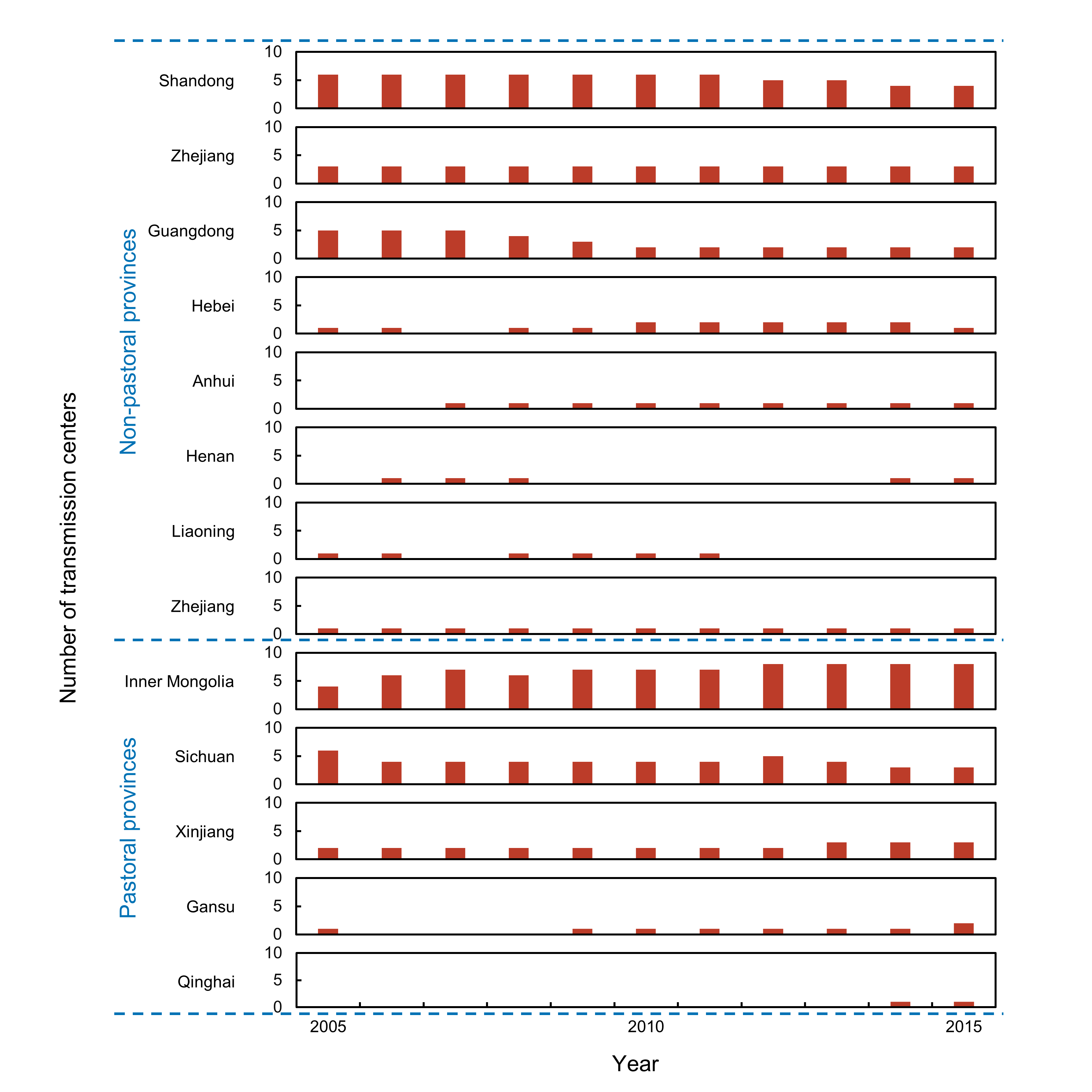


Fig. S2. Temporal variations in the number of transmission centers of forage-livestock conflicts in China in each province during 2005–2015.


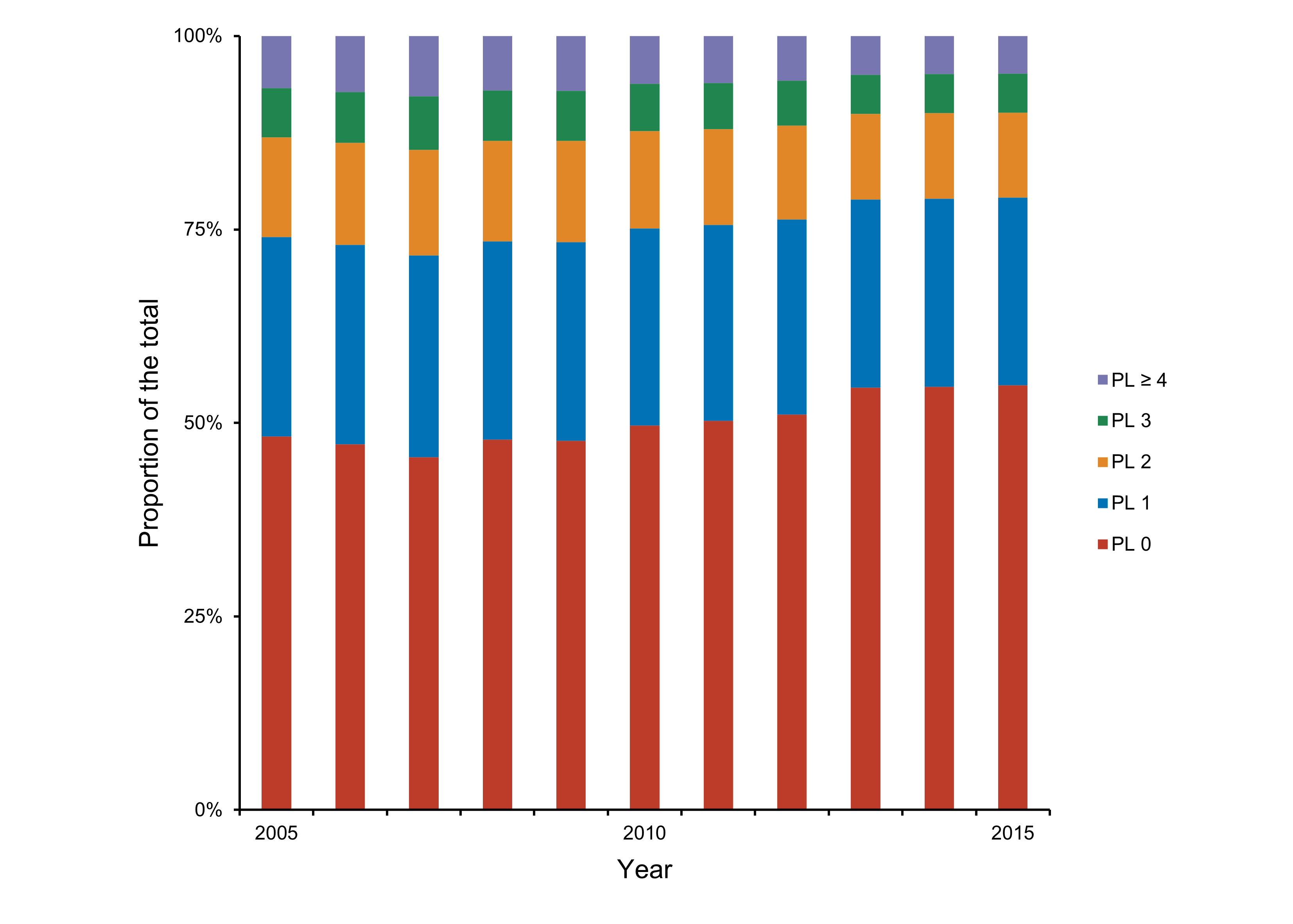


Fig. S3. Distribution of embodied forage-livestock conflicts in China at different production layers during 2005–2015. The abbreviation PL represents the production layer.


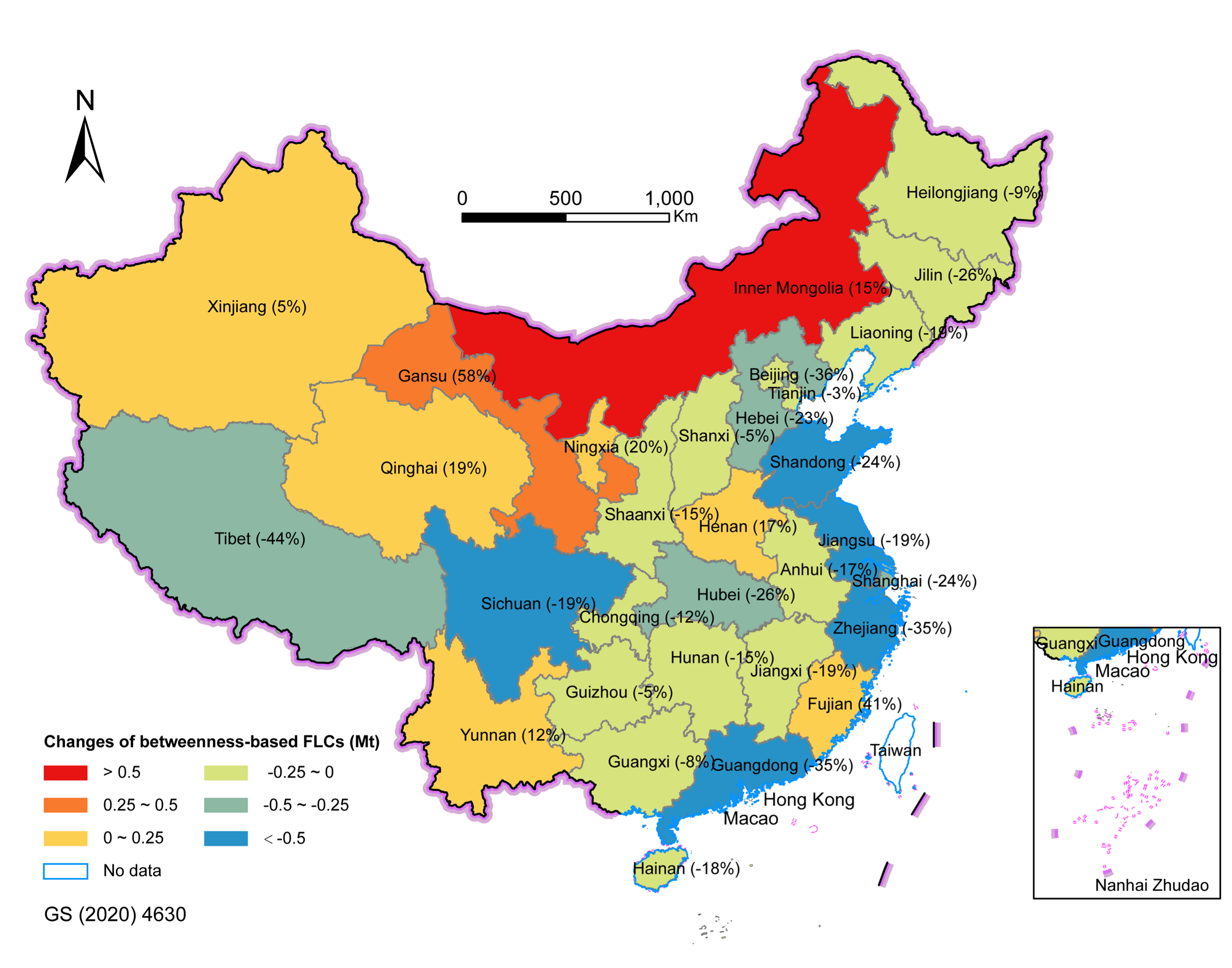


Fig. S4. Changes of betweenness-based forage-livestock conflicts in provinces during 2005–2015. The abbreviations FLCs and Mt are forage-livestock conflicts and million tons. The numbers in brackets are changing rates of betweenness-based forage-livestock conflicts in provinces from 2005 to 2015.

# Supplementary Tables

Table S1. The list of Chinese provinces included in this study

| **No.** | **Provinces** | **Abbreviations** |
| --- | --- | --- |
| 1 | Beijing | BJ |
| 2 | Tianjin | TJ |
| 3 | Hebei | HB |
| 4 | Shanxi | SX |
| 5 | Inner Mongolia | IM |
| 6 | Liaoning | LN |
| 7 | Jilin | JL |
| 8 | Heilongjiang | HLJ |
| 9 | Shanghai | SH |
| 10 | Jiangsu | JS |
| 11 | Zhejiang | ZJ |
| 12 | Anhui | AH |
| 13 | Fujian | FJ |
| 14 | Jiangxi | JX |
| 15 | Shandong | SD |
| 16 | Henan | HEN |
| 17 | Hubei | HUB |
| 18 | Hunan | HUN |
| 19 | Guangdong | GD |
| 20 | Guangxi | GX |
| 21 | Hainan | HAN |
| 22 | Chongqing | CQ |
| 23 | Sichuan | SC |
| 24 | Guizhou | GZ |
| 25 | Yunnan | YN |
| 26 | Tibet | \ |
| 27 | Shaanxi | SHX |
| 28 | Gansu | GS |
| 29 | Qinghai | QH |
| 30 | Ningxia | NX |
| 31 | Xinjiang | XJ |

Table S2. The concordance of sectors for MRIO tables in this study and that compiled by Wang et al.

| **No.** | **58sectors** | **Abbreviations** | **42sectors** |
| --- | --- | --- | --- |
| 1 | Agriculture | AGR | Agriculture, forestry, animal husbandry, and fishery |
| 2 | Forestry | FOR |
| 3 | Fishery | FISH |
| 4 | Rangeland-based livestock raising | RbLR |
| 5 | Confinement-based livestock raising | CbLR |
| 6 | Pig raising | PIG |
| 7 | Poultry raising | POU |
| 8 | Hunting and capturing animals | H&C |
| 9 | Other animal husbandry | OAH |
| 10 | Agriculture, forestry, animal husbandry, and fishery service | AGS |
| 11 | Coal mining and dressing | CM&D | Coal mining and dressing |
| 12 | Petroleum and natural gas extraction | P&NG | Petroleum and natural gas extraction |
| 13 | Metal ores mining and dressing | MM&D | Metal ores mining and dressing |
| 14 | Nonmetal and other minerals mining | NM&MM | Nonmetal and other minerals mining |
| 15 | Agricultural and sideline products processing | A&SP | Food processing and tobacco |
| 16 | Food manufacturing | FM |
| 17 | Beverage manufacturing | BM |
| 18 | Tobacco products manufacturing | TPM |
| 19 | Textile industry | TEX | Textile industry |
| 20 | Textile garments, footwear, caps, leather, etc. | TG&FCL | Textile garments, footwear, caps, leather, etc. |
| 21 | Timber processing and furniture manufacturing | TP&FM | Timber processing and furniture manufacturing |
| 22 | Paper printing, cultural, educational, and sports products | PCE&S | Paper printing, cultural, educational, and sports products |
| 23 | Petroleum processing, coking, and nuclear fuel processing | PC&NFP | Petroleum processing, coking, and nuclear fuel processing |
| 24 | Chemical raw materials and chemical products | CRM&CP | Chemical industry |
| 25 | Medicines | MED |
| 26 | Chemical fibre | CFI |
| 27 | Rubber and Plastic | R&P |
| 28 | Nonmetal mineral products | NMP | Nonmetal mineral products |
| 29 | Smelting and pressing of metals | S&PM | Smelting and pressing of metals |
| 30 | Metal products | MP | Metal products |
| 31 | General equipment manufacturing | GEM | General equipment manufacturing |
| 32 | Special equipment manufacturing | SEM | Special equipment manufacturing |
| 33 | Transportation equipment manufacturing | TEM | Transportation equipment manufacturing |
| 34 | Electrical machinery and equipment manufacturing | EM&EM | Electrical machinery and equipment manufacturing |
| 35 | Electronic equipment manufacturing | EEM | Electronic equipment manufacturing |
| 36 | Instrument and cultural office machinery manufacturing | I&COM | Instrument and cultural office machinery manufacturing |
| 37 | Other manufacturing | OM | Other manufacturing |
| 38 | Waster and flotsam | W&F | Waster and flotsam |
| 39 | Repair service for metal products, machinery, and equipment | REP | Repair service for metal products, machinery, and equipment |
| 40 | Production and supply of electric and heat power | E&H | Production and supply of electric and heat power |
| 41 | Production and supply of gas | GAS | Production and supply of gas |
| 42 | Production and supply of water | WAT | Production and supply of water |
| 43 | Construction | CON | Construction |
| 44 | Wholesale and retail | W&R | Wholesale and retail |
| 45 | Transport and storage | T&S | Transport and storage |
| 46 | Lodging | LOD | Hotel and restaurant |
| 47 | Catering | CAT |
| 48 | Information transfer and software | IT&S | Information transfer and software |
| 49 | Banking | BANK | Banking |
| 50 | Real estate trade | RET | Real estate trade |
| 51 | Leasing and commercial services | LEA | Leasing and commercial services |
| 52 | Research and experimental development | R&D | Research and experimental development |
| 53 | Management of water conservancy, environment, and public establishments | MAN | Management of water conservancy, environment, and public establishments |
| 54 | Residential services and other services | RES | Residential services and other services |
| 55 | Education | EDU | Education |
| 56 | Sanitation and social welfare | SOC | Sanitation and social welfare |
| 57 | Culture, sports, and entertainment | CS&E | Culture, sports, and entertainment |
| 58 | Public management and social organizations | PUB | Public management and social organizations |

Table S3. Datasets used in this study

| **No.** | **Data description** | **Sources** | **Data availability** |
| --- | --- | --- | --- |
| 1 | Administrative map | NCSFGI | <https://www.webmap.cn/commres.do?method=result100W> |
| 2 | Vegetation distribution map | [1] | <https://data.tpdc.ac.cn/en/data/> |
| 3 | Land use data | CNLUCC [2] | <https://www.resdc.cn/DOI/doi.aspx?DOIid=54> |
| 4 | Protected area distribution | ArcGIS Online | <http://120.26.232.88:6080/arcgis/rest/services/ROOT/HJMGQ/MapServer> |
| 5 | Net primary production | MODIS | <http://www.ntsg.umt.edu/> |
| 6 | Domestic livestock data | Provincial statistic yearbooks | <https://data.cnki.net/Yearbook/> |
|  |  | China animal husbandry and veterinary statistical yearbooks |  |
| 7 | Conversion factors of SSU of each type of livestock | [3], [4], [5] |  |
| 8 | Proper utilization ratio in each rangeland type | [3] |  |
| 9 | The ratio of aboveground to underground biomass in each rangeland type | [6], [7] |  |
| 10 | The proportion of forage and scrubs in each rangeland type | [7] |  |
| 11 | The proportion of edible forage in each rangeland type | [8] |  |
| 12 | The proportion of edible scrubs in each rangeland type | [7] |  |

Table S4. Temporal variations in the rankings of critical transmission centers in terms of betweenness-based forage-livestock conflicts in China during 2005–2015

| **No.** | **Provincial sectors** | **2005** | **2006** | **2007** | **2008** | **2009** | **2010** | **2011** | **2012** | **2013** | **2014** | **2015** |
| --- | --- | --- | --- | --- | --- | --- | --- | --- | --- | --- | --- | --- |
| 1 | Inner Mongolia - Agricultural and sideline products processing | 3 | 2 | 1 | 1 | 1 | 1 | 1 | 1 | 1 | 1 | 1 |
| 2 | Shandong - Agricultural and sideline products processing | 2 | 3 | 3 | 2 | 2 | 2 | 2 | 2 | 2 | 2 | 2 |
| 3 | Inner Mongolia - Agriculture | 8 | 5 | 6 | 8 | 6 | 5 | 5 | 6 | 3 | 3 | 3 |
| 4 | Inner Mongolia - Food manufacturing | 1 | 1 | 2 | 3 | 3 | 3 | 3 | 4 | 5 | 5 | 4 |
| 5 | Jiangsu - Textile industry | 4 | 4 | 4 | 4 | 4 | 4 | 4 | 3 | 4 | 4 | 5 |
| 6 | Sichuan - Agricultural and sideline products processing | 5 | 8 | 5 | 5 | 5 | 6 | 6 | 5 | 7 | 6 | 6 |
| 7 | Shandong - Textile industry | 6 | 6 | 8 | 6 | 7 | 7 | 7 | 9 | 6 | 7 | 7 |
| 8 | Xinjiang - Agriculture | 12 | 10 | 14 | 13 | 12 | 11 | 11 | 11 | 10 | 8 | 8 |
| 9 | Sichuan - Beverage manufacturing | 9 | 9 | 9 | 10 | 11 | 9 | 8 | 7 | 8 | 10 | 9 |
| 10 | Sichuan - Agriculture | 10 | 13 | 11 | 12 | 9 | 8 | 9 | 8 | 9 | 9 | 10 |
| 11 | Xinjiang - Agricultural and sideline products processing | 11 | 11 | 13 | 11 | 13 | 12 | 12 | 12 | 12 | 11 | 11 |
| 12 | Jiangsu - Agricultural and sideline products processing | 13 | 12 | 10 | 9 | 10 | 10 | 10 | 10 | 11 | 12 | 12 |
| 13 | Zhejiang - Textile industry | 7 | 7 | 7 | 7 | 8 | 13 | 13 | 13 | 13 | 13 | 13 |
| 14 | Gansu - Agriculture | 26 | 31 | 40 | 32 | 28 | 23 | 21 | 15 | 14 | 14 | 14 |
| 15 | Inner Mongolia - Beverage manufacturing | 34 | 21 | 20 | 22 | 17 | 16 | 15 | 14 | 15 | 15 | 15 |
| 16 | Shandong - Agriculture | 15 | 15 | 17 | 17 | 15 | 14 | 14 | 16 | 17 | 16 | 16 |
| 17 | Inner Mongolia - Rangeland-based livestock raising | 39 | 33 | 22 | 31 | 21 | 17 | 17 | 21 | 16 | 17 | 17 |
| 18 | Anhui - Agricultural and sideline products processing | 32 | 35 | 27 | 21 | 20 | 18 | 16 | 17 | 18 | 18 | 18 |
| 19 | Inner Mongolia - Confinement-based livestock raising | 33 | 23 | 26 | 23 | 22 | 21 | 24 | 18 | 19 | 20 | 19 |
| 20 | Guangdong - Textile industry | 14 | 14 | 12 | 14 | 14 | 20 | 20 | 20 | 20 | 19 | 20 |
| 21 | Guangdong - Paper printing, cultural, educational and sports products | 16 | 16 | 15 | 16 | 16 | 22 | 23 | 23 | 21 | 21 | 21 |
| 22 | Inner Mongolia - Lodging | 19 | 17 | 18 | 19 | 19 | 15 | 22 | 22 | 22 | 22 | 22 |
| 23 | Qinghai - Agricultural and sideline products processing | 37 | 61 | 55 | 43 | 50 | 60 | 43 | 44 | 38 | 24 | 23 |
| 24 | Shandong - Food manufacturing | 18 | 18 | 19 | 18 | 18 | 19 | 19 | 25 | 27 | 26 | 24 |
| 25 | Henan - Agriculture | 113 | 34 | 34 | 37 | 34 | 31 | 34 | 35 | 31 | 28 | 25 |
| 26 | Xinjiang - Food manufacturing | 36 | 36 | 41 | 35 | 36 | 34 | 38 | 38 | 26 | 23 | 26 |
| 27 | Hebei - Agriculture | 31 | 38 | 35 | 38 | 38 | 30 | 30 | 30 | 24 | 27 | 27 |
| 28 | Jiangsu - Chemical raw materials and chemical products | 22 | 20 | 21 | 20 | 23 | 24 | 25 | 24 | 25 | 25 | 28 |
| 29 | Inner Mongolia - Chemical raw materials and chemical products | 47 | 41 | 38 | 42 | 35 | 32 | 37 | 26 | 23 | 29 | 29 |
| 30 | Gansu - Agricultural and sideline products processing | 81 | 106 | 106 | 82 | 75 | 69 | 61 | 48 | 40 | 42 | 30 |
| 31 | Shandong - Timber processing and furniture manufacturing | 21 | 22 | 30 | 27 | 27 | 28 | 28 | 32 | 33 | 32 | 31 |
| 32 | Shandong - Chemical raw materials and chemical products | 27 | 24 | 28 | 26 | 25 | 27 | 27 | 29 | 29 | 31 | 32 |
| 33 | Hebei - Agricultural and sideline products processing | 20 | 29 | 31 | 25 | 26 | 26 | 26 | 28 | 28 | 30 | 33 |
| 34 | Liaoning - Agricultural and sideline products processing | 28 | 30 | 32 | 30 | 30 | 29 | 29 | 33 | 32 | 34 | 35 |
| 35 | Sichuan - Pig raising | 17 | 19 | 16 | 15 | 24 | 25 | 18 | 19 | 30 | 33 | 37 |
| 36 | Henan - Agricultural and sideline products processing | 104 | 26 | 25 | 29 | 33 | 36 | 36 | 36 | 34 | 35 | 39 |
| 37 | Sichuan - Textile industry | 29 | 37 | 33 | 36 | 39 | 33 | 31 | 27 | 37 | 39 | 40 |
| 38 | Sichuan - Timber processing and furniture manufacturing | 30 | 39 | 36 | 41 | 41 | 38 | 35 | 34 | 41 | 40 | 42 |
| 39 | Guangdong - Agricultural and sideline products processing | 24 | 27 | 24 | 24 | 29 | 39 | 40 | 39 | 43 | 41 | 44 |
| 40 | Guangdong - Textile garments, footwear, caps, leather, etc. | 25 | 28 | 29 | 34 | 32 | 43 | 44 | 46 | 46 | 47 | 46 |
| 41 | Guangdong - Chemical raw materials and chemical products | 23 | 25 | 23 | 28 | 31 | 42 | 41 | 45 | 47 | 46 | 47 |

Table S6. The *in-situ* forage-livestock conflicts induced by the grazing activities in each pastoral province during 2005–2015 (million tons)

| **No.** | **Provinces** | **2005** | **2006** | **2007** | **2008** | **2009** | **2010** | **2011** | **2012** | **2013** | **2014** | **2015** |
| --- | --- | --- | --- | --- | --- | --- | --- | --- | --- | --- | --- | --- |
| 1 | Inner Mongolia | 22.57 | 26.37 | 30.13 | 25.09 | 27.79 | 30.01 | 28.13 | 25.52 | 28.30 | 30.16 | 32.92 |
| 2 | Sichuan | 13.12 | 13.00 | 14.74 | 13.59 | 13.15 | 13.49 | 13.73 | 14.00 | 9.84 | 10.91 | 11.23 |
| 3 | Yunnan | 0.22 | 0.20 | 0.42 | 0.32 | 0.27 | 0.32 | 0.29 | 0.34 | 0.31 | 0.35 | 0.31 |
| 4 | Tibet | 16.03 | 15.52 | 16.18 | 15.12 | 14.57 | 12.69 | 11.78 | 10.99 | 10.41 | 10.33 | 9.48 |
| 5 | Gansu | 4.99 | 4.66 | 4.52 | 5.64 | 5.62 | 5.76 | 6.22 | 7.22 | 7.70 | 8.53 | 9.51 |
| 6 | Qinghai | 9.08 | 8.87 | 10.29 | 10.04 | 9.15 | 8.46 | 10.51 | 9.89 | 9.02 | 10.91 | 11.78 |
| 7 | Xinjiang | 10.95 | 11.85 | 11.17 | 11.66 | 10.72 | 11.02 | 11.34 | 10.47 | 11.78 | 13.54 | 14.10 |

Table S7. Betweenness-based forage-livestock conflicts of provinces in China during 2005–2015 (million tons)

| **Provinces** | **2005** | **2006** | **2007** | **2008** | **2009** | **2010** | **2011** | **2012** | **2013** | **2014** | **2015** |
| --- | --- | --- | --- | --- | --- | --- | --- | --- | --- | --- | --- |
| Beijing | 0.59 | 0.60 | 0.70 | 0.59 | 0.59 | 0.48 | 0.47 | 0.38 | 0.34 | 0.37 | 0.38 |
| Tianjin | 0.38 | 0.48 | 0.57 | 0.47 | 0.48 | 0.44 | 0.43 | 0.39 | 0.33 | 0.36 | 0.37 |
| Hebei | 1.88 | 1.82 | 2.14 | 1.81 | 1.80 | 1.63 | 1.60 | 1.49 | 1.30 | 1.40 | 1.45 |
| Shanxi | 0.56 | 0.61 | 0.71 | 0.60 | 0.60 | 0.56 | 0.54 | 0.51 | 0.47 | 0.50 | 0.53 |
| Inner Mongolia | 5.19 | 6.03 | 7.22 | 5.79 | 6.41 | 6.45 | 5.99 | 5.58 | 5.16 | 5.51 | 5.98 |
| Liaoning | 0.96 | 1.04 | 1.22 | 1.03 | 1.02 | 0.93 | 0.91 | 0.82 | 0.70 | 0.74 | 0.78 |
| Jilin | 0.53 | 0.57 | 0.68 | 0.59 | 0.59 | 0.53 | 0.52 | 0.40 | 0.35 | 0.38 | 0.39 |
| Heilongjiang | 0.46 | 0.49 | 0.57 | 0.50 | 0.50 | 0.47 | 0.46 | 0.42 | 0.36 | 0.40 | 0.42 |
| Shanghai | 1.35 | 1.48 | 1.74 | 1.46 | 1.45 | 1.30 | 1.27 | 1.05 | 0.92 | 1.00 | 1.03 |
| Jiangsu | 3.36 | 3.85 | 4.46 | 3.75 | 3.73 | 3.35 | 3.27 | 2.91 | 2.44 | 2.64 | 2.71 |
| Zhejiang | 2.10 | 2.29 | 2.68 | 2.21 | 2.20 | 1.38 | 1.35 | 1.37 | 1.21 | 1.32 | 1.36 |
| Anhui | 1.33 | 1.42 | 1.64 | 1.41 | 1.40 | 1.27 | 1.24 | 1.15 | 0.99 | 1.07 | 1.10 |
| Fujian | 0.46 | 0.96 | 1.10 | 0.95 | 0.94 | 0.84 | 0.82 | 0.69 | 0.58 | 0.62 | 0.65 |
| Jiangxi | 0.68 | 0.73 | 0.83 | 0.73 | 0.72 | 0.64 | 0.63 | 0.58 | 0.50 | 0.54 | 0.55 |
| Shandong | 4.73 | 4.95 | 5.41 | 4.82 | 4.80 | 4.39 | 4.30 | 3.82 | 3.25 | 3.50 | 3.61 |
| Henan | 0.96 | 1.39 | 1.62 | 1.38 | 1.37 | 1.17 | 1.15 | 1.07 | 0.97 | 1.07 | 1.12 |
| Hubei | 1.27 | 1.40 | 1.55 | 1.38 | 1.36 | 1.23 | 1.21 | 0.97 | 0.85 | 0.91 | 0.94 |
| Hunan | 0.75 | 0.81 | 0.92 | 0.80 | 0.79 | 0.72 | 0.70 | 0.65 | 0.53 | 0.62 | 0.64 |
| Guangdong | 3.07 | 3.29 | 3.94 | 3.24 | 3.22 | 2.37 | 2.31 | 2.11 | 1.78 | 1.93 | 1.99 |
| Guangxi | 0.49 | 0.52 | 0.60 | 0.52 | 0.53 | 0.52 | 0.52 | 0.48 | 0.40 | 0.43 | 0.45 |
| Hainan | 0.11 | 0.12 | 0.14 | 0.12 | 0.12 | 0.11 | 0.11 | 0.10 | 0.08 | 0.09 | 0.09 |
| Chongqing | 0.41 | 0.47 | 0.50 | 0.48 | 0.47 | 0.42 | 0.41 | 0.38 | 0.33 | 0.35 | 0.36 |
| Sichuan | 4.30 | 4.22 | 5.17 | 4.38 | 4.25 | 4.27 | 4.34 | 4.34 | 3.05 | 3.37 | 3.47 |
| Guizhou | 0.19 | 0.21 | 0.22 | 0.21 | 0.21 | 0.19 | 0.19 | 0.17 | 0.16 | 0.17 | 0.18 |
| Yunnan | 0.43 | 0.46 | 0.56 | 0.48 | 0.48 | 0.46 | 0.45 | 0.43 | 0.40 | 0.46 | 0.48 |
| Tibet | 0.73 | 0.71 | 0.73 | 0.69 | 0.68 | 0.55 | 0.51 | 0.50 | 0.42 | 0.42 | 0.41 |
| Shaanxi | 0.72 | 0.77 | 0.89 | 0.77 | 0.76 | 0.71 | 0.70 | 0.64 | 0.54 | 0.59 | 0.61 |
| Gansu | 0.77 | 0.75 | 0.80 | 0.86 | 0.85 | 0.84 | 0.88 | 0.99 | 0.99 | 1.10 | 1.22 |
| Qinghai | 1.06 | 1.05 | 1.42 | 1.17 | 1.08 | 0.94 | 1.14 | 1.07 | 0.96 | 1.17 | 1.26 |
| Ningxia | 0.05 | 0.06 | 0.06 | 0.06 | 0.06 | 0.06 | 0.05 | 0.05 | 0.05 | 0.06 | 0.06 |
| Xinjiang | 2.00 | 2.17 | 2.18 | 2.14 | 1.98 | 1.96 | 2.00 | 1.81 | 1.73 | 2.00 | 2.10 |

# Supplementary Methods

We compiled the Chinese forage-livestock conflict inventory by subtracting the available forage supply from the total forage demand of herbivores in rangelands. First, we overlaid the land use map, vegetation distribution map, protected area map, and administrative map to derive the scope of available natural rangelands in pastoral provinces of China. Second, we employed the rangeland yield model to calculate grid-level available forage supply. Third, we converted different types of livestock into standard sheep units to estimate county-level forage demand. Fourth, we used the zonal statistic method to get provincial forage-livestock conflicts. Meanwhile, we applied a Monte Carlo simulation with 10,000 samplings to evaluate the uncertainty range of FLCs of each province. The framework of the compilation of the Chinese forage-livestock conflict inventory is illustrated in Fig. S5.


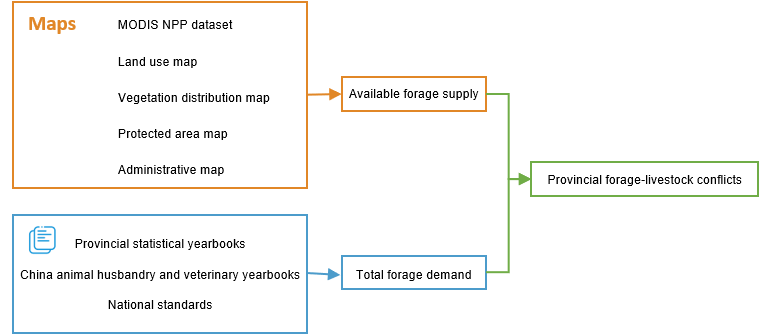


Fig. S5. The framework of the compilation of the Chinese forage-livestock conflict inventory in this study.

**Scope of rangelands in pastoral China**

There are 268 official pastoral counties in China, which are located in 13 provinces. We selected 229 pastoral counties in Inner Mongolia, Xinjiang, Tibet, Qinghai, Gansu, Sichuan, and Yunnan provinces as the pastoral regions of China because the remaining 39 pastoral counties in the other 6 provinces (Shanxi, Hebei, Heilongjiang, Liaoning, Jilin, and Ningxia) had gradually implemented the Grazing Prohibition Policy on rangelands since 2001.

We derived the scope of natural rangelands in the pastoral regions of China based on China’s Multi-Period Land Use Land Cover Remote Sensing Monitoring Dataset [2] (CNLUCC) and the vegetation distribution map of China [1]. Following the approach proposed by Alkemade et al. [9], we used CNLUCC to select land cover types for rangelands, which include grassland, desert, marsh, and shrubland. We further used the vegetation distribution map to restrict our areas to natural rangelands. The vegetation types selected in this study include (1) alpine vegetation, (2) (semi-) deserts, (3) grasslands, (4) meadows, (5) high-cold marshes and cold-temperate and temperate marshes, (6) scrubs, and (7) steppes. Moreover, we excluded the core regions of protected areas because of the prohibition of pastoralism in these regions. The CNLUCC dataset is only available for 2005, 2010, and 2015. We assumed that the land cover does not change significantly and remains constant every five years (i.e., for 2005–2007 it's 2005 data, for 2008–2012 it's 2010 data, and for 2013–2015 it's 2015 data).

**Estimation of available forage supply**

We employed the rangeland yield model of Su et al. [3] to estimate the available forage supply. The rangeland yield model can assess the forage resources at the grid level and is commonly used in China's rangeland resource assessment studies [10], [11], [5], [12]. The estimation of available forage supply mainly depends on net primary production (NPP) and the rangeland type. We used the MOD 17 dataset from the Moderate-resolution Imaging Spectroradiometer (MODIS) as the NPP source. We generated the rangeland-type data from the vegetation distribution map. The estimation of grid-level available forage supply can be calculated as follows:

$FY_{n}^{\text{k}}=\sum_{i}^{n} \frac{NPP_{\text{i}}^{k}\times U_{i}^{k}\times\text{（}E_{i}^{k}\times P_{i}^{k}+ES_{i}^{k}\times PS_{i}^{k})}{\text{t（1+r}_{i}^{k}\text{）}}$ (1)

where $FY_{n}^{k}$ represents the available forage supply of natural rangeland of county *n* in year *k*. The notions $U_{i}^{k}$, $P_{i}^{k}$, $PS_{i}^{k}$, $E_{i}^{k}$, $ES_{i}^{k}$, and $r_{i}^{k}$ are the proper utilization rate [3], the proportion of forage [7], the proportion of scrubs [7], the proportion of edible forage [8], the proportion of edible scrubs [7], and the ratio of aboveground to underground biomass [6] of rangeland vegetation of grid *i* in year *k*, respectively. We assumed that $U_{i}^{k}$, $P_{i}^{k}$, $PS_{i}^{k}$, $E_{i}^{k}$, $ES_{i}^{k}$, and $r_{i}^{k}$ have the same value in a certain rangeland type due to the lack of more accurate spatial heterogeneity data. The notation *t* is the coefficient of biomass conversion to productivity, we set 0.45 here according to a previous study [13].

**Estimation of total forage demand**

Total forage demand includes forage demand by domestic livestock and wild herbivores. We converted different types of domestic livestock into the standard sheep unit (SSU), which is defined as a 50 kg adult sheep that consumes 1.8 kg dry matter (DM) forage per day. The conversion factors were derived from previous literature [3], [4], [14]. Due to the lack of high-resolution wild herbivores population data, we assumed that 10% of the available forage supply in each grid is consumed by wild herbivores based on literature sources [15], [16]. Therefore, the total forage demand in each county is estimated as eq 2:

$FD_{n}^{k}=I_{d}\times\text{D}\times\sum_{j} \text{（LO}_{n,j}^{k}\times\alpha_{j}\text{+LS}_{n,j}^{k}\times\beta_{j}\text{）}+\sum_{i} FDW_{n,i}^{k}$ (2)

where $FD_{n}^{k}$ is the total forage demand of county *n* in year *k*; I_d_ is the daily intake per SSU, set to 1.8 kg DM per day; D is the number of grazing days, which is 365 in this study. The notations $LO_{n,j}^{k}$and $LS_{n,j}^{k}$ are the number of livestock *j* in stock and slaughtered of county *n* in year *k*, respectively. The notations $\alpha_{j}$ and $\beta_{j}$ are conversion factors of SSU of livestock *j* in stock and slaughtered, respectively. The notation $FDW_{n,i}^{k}$ is the forage demand by wild herbivores in grid *i* of county *n* in year *k*. Data for county-level domestic livestock data are obtained from China Animal Husbandry and Veterinary Yearbooks [17] and provincial statistical yearbooks [18], [19], [20], [21], [22], [23] published from 2006 to 2016.

# Supplementary References

[1] X. Hou, 1:1,000,000 scale vegetation distribution map of China, (2001).

[2] X. Xu, J. Liu, S. Zhang, R. Li, C. Yan, S. Wu, China’s Multi-Period Land Use Land Cover Remote Sensing Monitoring Dataset, (2018).

[3] D. Su, Z. Yang, X. Yun, Calculation of rangeland carrying capacity (NY/T 635-2015), (2015).

[4] Y. Dong, Q. Zhao, R. Na, F. Mao, W. Liu, L. Wang, E. Zhao, J. Wang, Q. Zhao, H. Zhang, X. Wu, W. Yang, Sheep unit conversion of grass-fed livestock (NY/T 3647-2020), (2020).

[5] T. Yang, J. Dong, L. Huang, Y. Li, H. Yan, J. Zhai, J. Wang, Z. Jin, G. Zhang, A large forage gap in forage availability in traditional pastoral regions in China, Fundamental Research. (2023). https://doi.org/10.1016/j.fmre.2023.01.003.

[6] Y. Sun, Y. Yang, X. Zhao, Z. Tang, S. Wang, J. Fang, Global patterns and climatic drivers of above- and belowground net primary productivity in grasslands, Sci. China Life Sci. 64 (2020) 739–751. https://doi.org/10.1007/s11427-020-1837-9.

[7] X. Yang, Y. Guo, M. Anniwaer, H. Liu, W. Ma, S. Yu, Z. Tang, Distribution of biomass in relation to environments in shrublands of temparate China, Chinese Journal of Plant Ecology. 41 (2017) 22–30.

[8] X. Liu, Q. Feng, T. Liang, R. Long, Spatial-temporal dynamic balance between livestock carrying capacity and productivity of rangeland in Gannan of Gansu Province, China., Chinese journal of grassland. 32 (2010) 99–106.

[9] R. Alkemade, R.S. Reid, M. van den Berg, J. de Leeuw, M. Jeuken, Assessing the impacts of livestock production on biodiversity in rangeland ecosystems, Proceedings of the National Academy of Sciences. 110 (2013) 20900–20905. https://doi.org/10.1073/pnas.1011013108.

[10] GEOARC, Eurasia Grassland Ecological Status, National Remote Sensing Center of China, Beijing, China, 2021.

[11] L. Huang, J. Ning, P. Zhu, Y. Zheng, J. Zhai, The conservation patterns of grassland ecosystem in response to the forage-livestock balance in North China, J. Geogr. Sci. 31 (2021) 518–534. https://doi.org/10.1007/s11442-021-1856-6.

[12] H. Yu, G. Wang, Y. Yang, Z. Bai, B. Liu, T. Zhang, Y. Xu, Y. Lu, Enhancing ecological value through sustainable food supply of grasslands in the Three-River-Source National Park, Tibet Plateau, China, Ecosystem Services. 46 (2020) 101218. https://doi.org/10.1016/j.ecoser.2020.101218.

[13] J. Chen, W. Fan, D. Li, X. Liu, M. Song, Driving factors of global carbon footprint pressure: Based on vegetation carbon sequestration, Applied Energy. 267 (2020) 114914. https://doi.org/10.1016/j.apenergy.2020.114914.

[14] N. Meng, L. Wang, W. Qi, X. Dai, Z. Li, Y. Yang, R. Li, J. Ma, H. Zheng, A high-resolution gridded grazing dataset of grassland ecosystem on the Qinghai–Tibet Plateau in 1982–2015, Sci Data. 10 (2023) 68. https://doi.org/10.1038/s41597-023-01970-1.

[15] K. Petz, R. Alkemade, M. Bakkenes, C.J.E. Schulp, M. van der Velde, R. Leemans, Mapping and modelling trade-offs and synergies between grazing intensity and ecosystem services in rangelands using global-scale datasets and models, Global Environmental Change. 29 (2014) 223–234. https://doi.org/10.1016/j.gloenvcha.2014.08.007.

[16] J. Chang, P. Ciais, M. Herrero, P. Havlik, M. Campioli, X. Zhang, Y. Bai, N. Viovy, J. Joiner, X. Wang, S. Peng, C. Yue, S. Piao, T. Wang, D.A. Hauglustaine, J.-F. Soussana, A. Peregon, N. Kosykh, N. Mironycheva-Tokareva, Combining livestock production information in a process-based vegetation model to reconstruct the history of grassland management, Biogeosciences. 13 (2016) 3757–3776. https://doi.org/10.5194/bg-13-3757-2016.

[17] China Animal Husbandry and Veterinary Statistical Yearbook Editorial Committee, China Animal Husbandry and Veterinary Statistical Yearbook, China Agricultural Press, Beijing, 2006.

[18] Inner Mongolia Municipal Bureau of Statistics, Inner Mongolia Statistical Yearbook, China Statistics Press, Beijing, 2006.

[19] Gansu Municipal Bureau of Statistics, Gansu Statistical Yearbook 2006, China Statistics Press, Beijing, China, 2006.

[20] Qinghai Municipal Bureau of Statistics, Qinghai Statistical Yearbook, China Statistics Press, Beijing, 2006.

[21] Sichuan Municipal Bureau of Statistics, Sichuan Statistical Yearbook, China Statistics Press, Beijing, 2006.

[22] Yunnan Municipal Bureau of Statistics, Yunnan Statistical Yearbook, China Statistics Press, Beijing, 2006.

[23] Xinjiang Municipal Bureau of Statistics, Xinjiang Statistical Yearbook, China Statistics Press, Beijing, 2006.
